# Dual-phase optimized deep learning framework for accurate, efficient, and robust battery SoC estimation

**DOI:** 10.1038/s41598-025-29449-6

**Published:** 2025-12-05

**Authors:** Sasikala R, Geetha Mani

**Affiliations:** School of Electrical Engineering, Vellore Institute of Technology, Vellore, Tamil Nadu 632014 India

**Keywords:** Deep learning, State of charge, Optuna, NSGA, Li-ion battery, Kolmogorov-Arnold network, Long short-term memory, Energy science and technology, Engineering, Materials science, Mathematics and computing

## Abstract

The growing adoption of electric vehicles (EVs) requires accurate and robust State of Charge (SoC) estimation to ensure optimal battery performance, reliable driving range, and operational safety. This paper introduces KANBiLSTMAtt, a novel hybrid deep learning model that integrates the Kolmogorov–Arnold Network (KAN), Bi-directional Long Short-Term Memory (BiLSTM), and attention mechanisms to capture nonlinear interactions and long-term temporal dependencies in lithium-ion battery data. The framework incorporates Optuna for efficient hyperparameter tuning and NSGA-II for multi-objective optimization, achieving high predictive accuracy with minimal computational overhead. Validation on two distinct battery chemistries under varying temperatures, using the LG dataset and driving cycles from the CALCE dataset, demonstrates strong generalization and robustness. KANBiLSTMAtt achieves an RMSE of 0.02%, a MAE of 0.01%, and an R² of 99% for both datasets, utilizing a lightweight architecture and converging within 90 s, making it highly suitable for real-time and embedded battery management systems. By combining hybrid deep learning and evolutionary optimization, the proposed model addresses limitations of traditional SoC estimation methods, offering a scalable solution for next-generation EV energy management.

## Introduction

With the growing awareness of the energy crisis, climate change, and environmental pollution, electric vehicles are increasingly being recognized as a cleaner, more reliable, and future-oriented alternative to traditional internal combustion engine vehicles^1^. The cost, reliability, and restricted operational range are key issues that hold back a swift switch to electric vehicles. Most individuals are of the view that electric cars are a greener, more reliable, and more innovative alternative to traditional gasoline vehicles since environmental issues such as pollution, climate change, and the energy crisis are more highly profiled.

In electric vehicles, batteries serve as energy storage devices. Currently, lead acid, nickel-cobalt, nickel-cadmium, and lithium-ion batteries are used for this purpose. Among these, lithium-ion (Li-ion) batteries are emerging as the most popular energy storage solution for a wide range of applications, including electric vehicles, renewable energy sources, and portable electronic devices. This preference is due to their elevated energy density, prolonged cycle life, and low self-discharge rate^2^. Most individuals are of the view that electric cars are a greener, more reliable, and more innovative alternative to traditional gasoline vehicles since environmental issues such as pollution, climate change, and the energy crisis are more highly profiled.

The battery pack is typically the most expensive discrete part of a typical setup, accounting for about 35% to 45% of the overall manufacturing cost of an EV. As a result, ensuring the reliability and safety of the battery requires the implementation of a sophisticated Battery Management System (BMS). The BMS is responsible for managing the battery to enhance its performance and lifespan. It functions as a controller, consisting of both hardware and software. Key roles of the BMS include state estimation (such as State of Charge (SoC) and State of Health (SoH), cell balancing, protecting the battery from overcharging and deep discharging, thermal management, and facilitating communication with other devices, such as the Electronic Control Unit (ECU) and user interfaces. Among all the BMS functions, SoC estimation is the most critical and fundamental^3^ .

The SoC is described as the proportion of a battery’s remaining capacity to its rated capacity under specific conditions. This value ranges from 0% when the battery is completely discharged to 100% when it is fully charged, indicating the current energy available in the battery. Estimating the SoC is crucial not only for determining the battery’s available energy, but also for determining the battery’s lifetime significantly. SoC estimation represents the remaining battery capacity or driving range of the vehicle. Accurate, error-free estimation of SoC is required for ensuring the reliability, efficiency, and safety of Li-ion batteries. SoC estimation plays a crucial role in battery management systems, enhancing battery performance, avoiding overcharging or over-discharging, ensuring safety, and ultimately extending battery life. Since the SoC cannot be directly measured, it must be inferred from variables such as voltage, current, and temperature using various estimation methods. Estimating the SoC is particularly challenging due to the non-linear electrochemical behaviour of Li-ion batteries, and their performance is also affected by factors such as aging, temperature, and charge/discharge current.

Traditional methods are Coulomb Counting, Open Circuit Voltage (OCV) method, and Kalman Filter method (KF)^4^. The Coulomb has cumulative error challenges. OCV is not ideal for real-time applications like electric vehicles, since the method is employed only when the vehicles are stationary. The Kalman filter needs an exact battery design with its parameters. The data-driven approach is utilized to address these problems. Data data-driven approach requires measured data in the past, and it does not involve the battery model. Data-driven approaches are deep learning algorithms and machine learning models. Machine learning techniques do not impose an electrical or chemical model on the battery^5^. However, popular machine learning models like Support Vector Machine for regression (SVR) and Random Forest are not ideal for batteries due to their non-linear behaviour and inability to handle high-dimensional data, as well as their limitations in learning architecture. Deep learning models, on the other hand, can accurately capture battery behaviours through multi-layer neurons and nonlinear transformations^6^. A deep neural network (DNN) with multiple layers and backpropagation (BP) can learn complex data patterns. Battery SoC estimation is influenced not only by current measurement data but also by historical data. Deep learning (DL) algorithms are extensively used in various electric vehicle (EV) applications, including energy management, charging demand prediction, state of health (SoH) estimation^6–9^, fault detection^10^, cell balancing^11^, and thermal management.

Due to its ability to memorize previous inputs, a Recurrent Neural Network is preferred for sequential data analysis^12^. RNNs are especially applied to tasks involving temporal dynamics since, unlike standard neural networks, they have a feedback system that allows for remembering how things were previously. One of the problems with vanilla RNNs is vanishing gradients, which disallow them from learning long-term dependencies^13^. To circumvent this problem, complex models such as GRUs (Gated Recurrent Units) and LSTMs (Long Short-Term Memory) are employed. To preserve long-term dependencies, LSTM networks possess three main gates that manage information flow through the network^14^. Following input data, the DDNN reshapes and alters its architecture, varying the number of neurons or connections as required. It is applied in real-time learning, where new inputs make the network structure modify itself. Three essential gates control the data flow in the network so that long-term dependencies are preserved.

Deep learning methods for estimating SoC in lithium-ion batteries (LIBs) generally come under four broad categories: feed-forward neural networks (FNNs), recurrent neural networks (RNNs), convolutional neural networks (CNNs), and hybrid models^15,16^. FNNs, involving multiple hidden layers, are designed to map battery input data directly to respective SoC values^17,18^. To deal with the time-dependent nature of battery data, RNNs—more specifically, long short-term memory (LSTM) and gated recurrent unit (GRU) models—are preferably employed, as they capture the temporal patterns in measurements effectively^19,20^. For even superior performance, bidirectional versions such as BiLSTM and BiGRU examine the data in both forward and reverse directions, providing a more holistic perspective of the temporal sequence^21^.

RNNs have been augmented with attention mechanisms to allow the models to concentrate on the most informative time steps, particularly beneficial for multi-step SoC prediction. Convolutional models such as Temporal Convolutional Networks (TCNs) and 1D CNNs have been employed to capture essential time-related features from the data. Hybrid RNN-CNN models have been proposed to combine the feature extraction strength of CNNs and the sequential data processing ability of RNNs to provide better accuracy in SoC estimation^22^. In addition, some methods combine RNNs with filtering methods to suppress the impact of noise in sensor measurements. Recently, efficient transformer models—involving self-attention and feed-forward layers—have gained immense popularity as they can capture both long-range dependencies and important local patterns and hence are uniquely beneficial for state-of-charge prediction^23,24^.

In order to enhance the efficiency and effectiveness of training deep learning models, optimization methods play a vital role^25^. They assist in minimizing the loss, improving model generalizability. Methods such as Adam and Stochastic Gradient Descent (SGD) are popularly used to optimize model parameters, improving the speed of convergence and reducing computational expenses^26^. By preventing local minima, the performance is enhanced by powerful optimization methods. Adaptive methods like RMSprop, Adam, and Adagrad enhance training performance by individually varying learning rates during training. Adding regularization to these methods enhances generalization and minimizes overfitting.

**Table 1:** A brief literature survey of Li-ion battery SoC Estimation methods.

Reference	Applied methodology	Key benefits	Primary limitations
**Model-based approaches**			
^27^	IC curve methodology	Easy implementation	Very sensitive to noise
^28^	Adaptive unscented Kalman Filter combined with adaptive extended Kalman Filter and state-of-charge estimation matching techniques	Resilient to unknown initial SoC, robust under measurement noise	Accuracy depends on modelling precision; high computational demand
**Hybrid methodologies**			
^29^	Adaptive Kalman filter integrated with deep neural network architectures- TCN, GRU- MHA	Robust against noise and unknown initial SoC values	Complex architecture requires high computation time
^30^	Combined deep learning models (TCN + RNN(LSTM)) with PSO, Kalman filter, filter the noise in the output stage	Captures spatial and temporal characteristics; noise reduction via filtering	May suffer from estimation errors over long-term use or initial deviations
^31^	Fractional-order modified moving horizon for estimating SoC and a predictive control algorithm for optimizing current sequences	Fast convergence	Nonlinear dynamics not fully captured; limited SOP estimation accuracy
^32^	Coulomb counting methodology integrated with battery modelling and bottom-up optimization approaches	Accounts for environmental conditions and the driver’s behaviour	Requires precise initial SoC; current measurement errors accumulate
**Data-driven methodologies**			
^33^	Fuzzy logic systems combined with artificial neural network architectures	Subtractive clustering neuro-fuzzy system effectively estimates SoC	Needs precise fuzzy rules and substantial training data
^34^	Random forest regression methodologies	Handles diverse battery datasets	Risk of local minima; weak global exploration
^35^	Machine learning - control variable model approach	Data augmentation for small datasets; improved accuracy	Sensitive to missing sensors and data quality; short trips excluded
^36^	Recurrent neural network (RNN) architectures	Estimates SoC without accurate initial values	Generalization across diverse conditions has not been validated
^37,38^	Gated recurrent unit (GRU) and long short-term memory (LSTM) recurrent neural network architectures	Captures long temporal dependencies	Limited evaluation metrics (e.g., R² not reported)
^39^	LSTM-GRU hybrid architecture combined with LSTM-multiple linear regression fusion techniques	Uses real-world trip data; performs well across temperatures	Requires multiple input sources (voltage, current, motor speed, etc.); validated only on driving cycles
^40^	GPR (Gaussian Process Regression), NN	Light-weighted offline training process for estimating online SoC with high accuracy	Lack of robustness of generalizability across varying thermal environments.
^41^	SVD-AUPF CNN-BiLSTM	A dynamic, condition-oriented, data-driven method accurately co-estimates SoC and capacity, enabling online diagnostics of battery aging for cells.	Without timely updates, the capacity can fail to precisely track the SoC and compensation of temperature influence under dynamic conditions.

As shown in Table 1, model-based approaches are simple and interpretable but often suffer from noise sensitivity and strong dependency on accurate modelling. Data-driven methods, including ANN, LSTM, and GRU variants, can capture nonlinearities and temporal dependencies, yet they typically demand heavy computation and lack robustness. Hybrid approaches combine model-based and data-driven techniques to improve accuracy, but their architectures remain complex and prone to high training costs. In contrast, our proposed hybrid deep learning framework with dual-phase optimization unifies nonlinear feature extraction and temporal sequence learning while systematically reducing training complexity. This allows for accurate, efficient, and robust SoC estimation, addressing the limitations observed in prior studies. Although the proposed approach demonstrates promising results, the R² score, a key metric for evaluating the accuracy and explanatory power of estimation models, is notably absent in most of the analysis. Including this metric would provide a more comprehensive and robust assessment of the model’s performance. This research encompasses deep learning approaches and hybrid methodologies, including the Kolmogorov-Arnold network, bidirectional LSTM (Bi-LSTM), and the attention mechanism method with a dual optimization algorithm. The assessment utilized data from the LG and CALCE battery dataset, focusing on parameters such as voltage, current, and temperature. The main contribution of this paper is summarized as follows:

1. The proposed model is to improve the accuracy of the SoC estimation. Extract the structured representation from input data. Then captures the temporal dependencies in both forward and backward directions. Finally, the output is focused on the most important time steps.
2. The optimization method used to optimize the model performance by learning rate, batch size, number of hidden layers in the model, number of KAN units, and number of knots for the spline.
3. To minimize the SoC estimation error by considering, to reduce the training time, model size, and reduce the memory usage by NSGA-II.
4. The proposed method combines the advantages of hybrid optimization for the developed deep learning models to estimate the SoC accurately.

The structure of this paper is organized as follows. Section II presents the architecture of the proposed deep learning model, detailing its design and functionality. Section III describes the optimization algorithm integrated into the framework to improve model efficiency and accuracy. Section IV outlines the dataset characteristics and the methodology adopted for hyperparameter selection. Section V defines the performance metrics used to evaluate the accuracy of State of Charge (SoC) estimation. Section VI explains the input features utilized in the model. Section VII discusses the results obtained, along with an in-depth analysis and interpretation. Finally, Section VIII concludes the study by summarizing the key findings and suggesting potential directions for future work.

## Deep learning model structure of the method proposed

### KAN

The Kolmogorov-Arnold Network (KAN) is based on the Kolmogorov-Arnold Representation Theorem, which states that any continuous multivariable function can be expressed as a finite sum of single-variable functions applied to linear combinations of the input variables^42^. For any continuous function f() as,

1 $$\:f\left({x}_{1},{x}_{2},\dots\:{x}_{n}\right)={\sum\:_{a=0}^{2n}{\phi}_{a}\left({{\sum\:}_{b=1}^{n}{\lambda\:}_{ab}{x}_{b}}_{.}\right)}_{.}$$

In Eq. 1, ф_a_ are continuous univariate functions and λ_ab_ are coefficients that are fixed and represent the linear transformations of input variables. KAN is composed of several layers of trainable univariate functions, rather than using traditional activation functions like ReLU or sigmoid. Such functions tend to be parameterized by splines or other functional approximators. For an input vector I ε Rn, KAN applies a structured transformation as,

2 $$\:{hd}_{i}^{\left(l\right)}={{\phi}}_{i}^{\left(l\right)}({w}_{i}^{\left(l\right)}I+{b}_{i}^{\left(l\right)})$$

In Eq. 2, l is the layer index, Wi is a learnable weight matrix b_i_ is a bias term, and ф_i_ is a learnable univariate function, often represented by spline interpolation or bias functions.

**Fig. 1:**
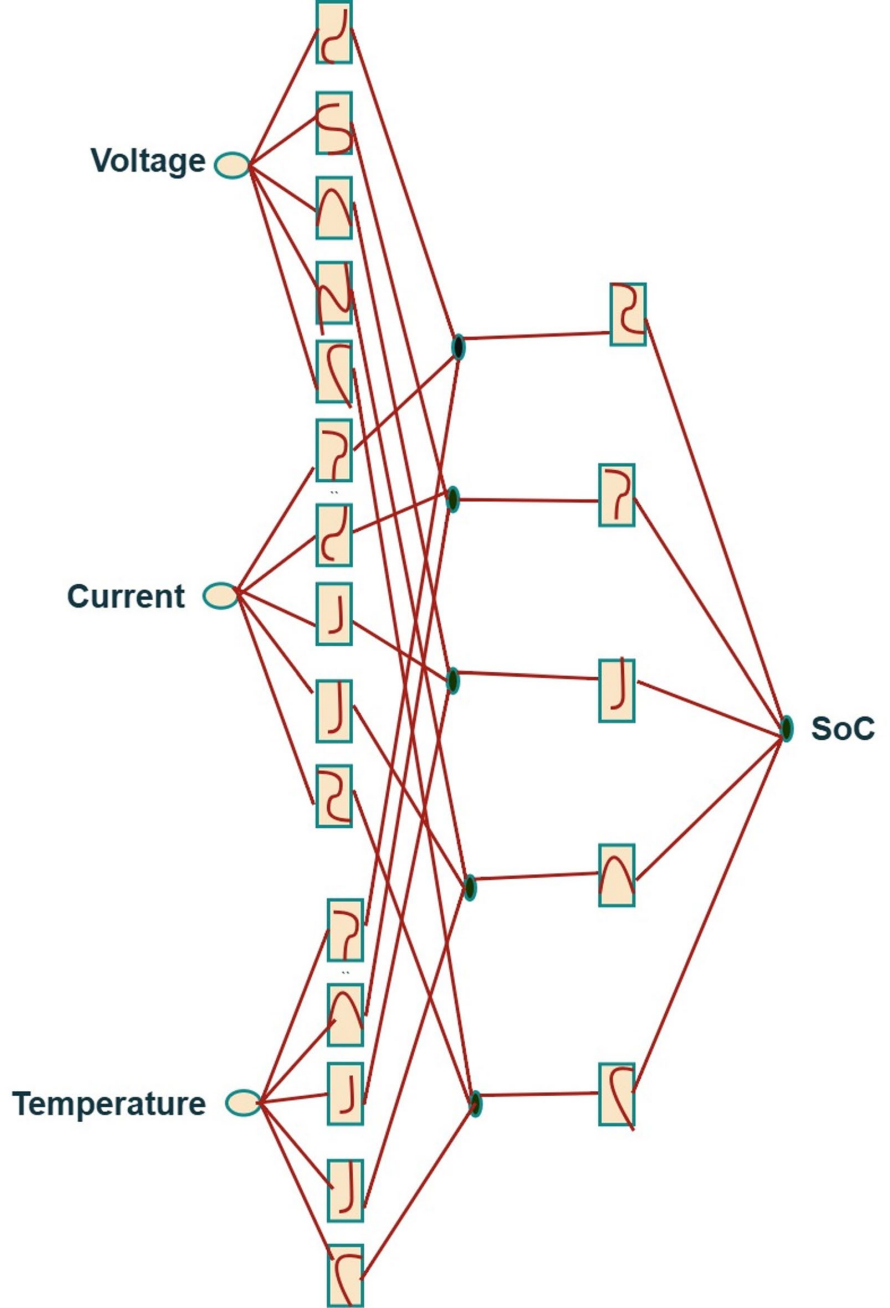
Kolmogorov-Arnold network for SoC estimation.

The Kolmogorov-Arnold network customized for SoC estimation is shown in Fig. 1. In this, the inputs are voltage, current, and temperature. Splines are used here instead of activation functions. In this continuous multivariable function, voltage, current, and temperature can be expressed as a finite sum of single-variable functions applied to linear combinations of the input variables. SoC is the output of the network.

### LSTM

**Fig. 2:**
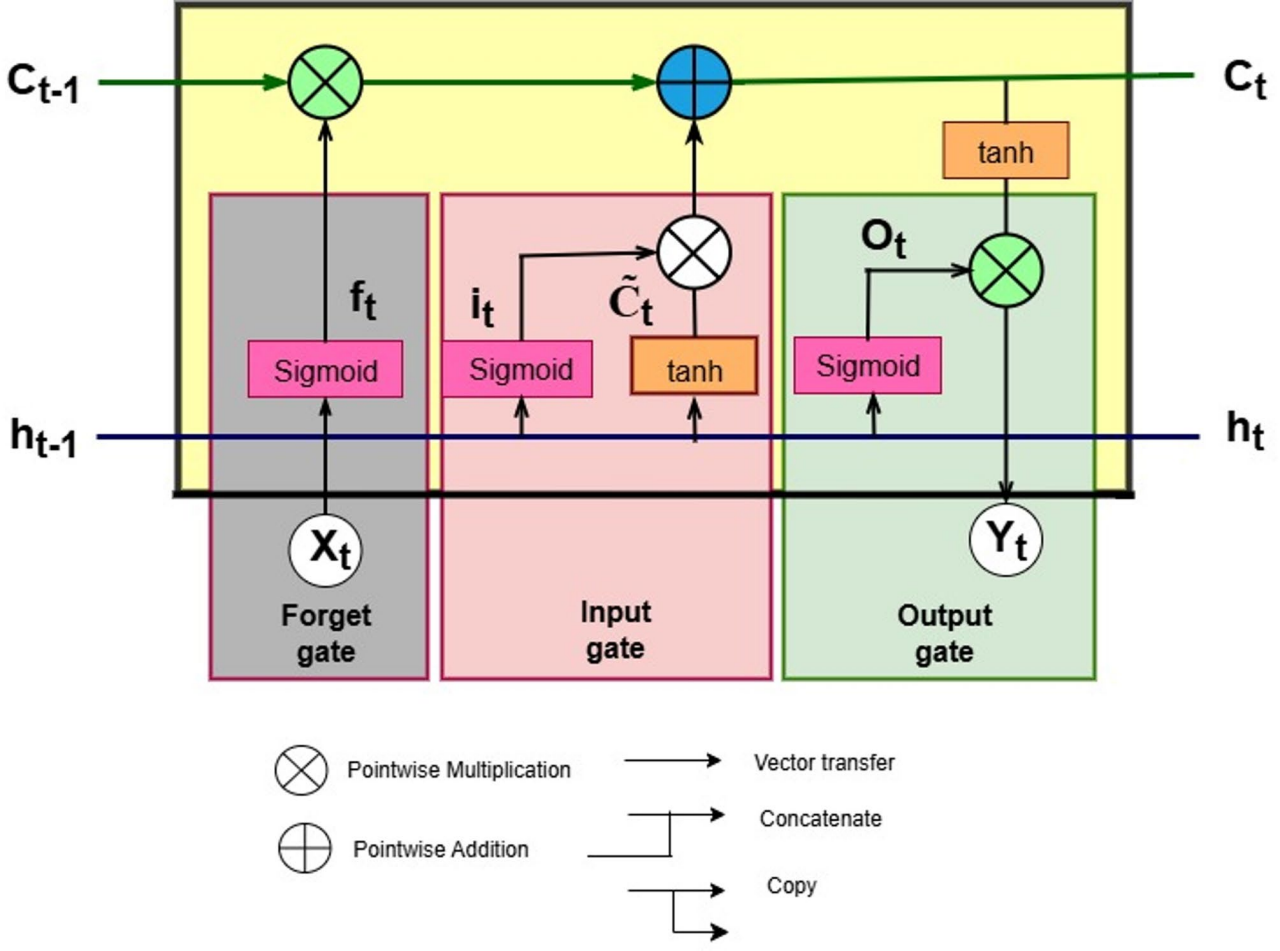
LSTM architecture.

LSTM (Long Short-Term Memory) resolves the vanishing/ exploding gradient problem in RNN, specifically in long sequence data^43^. It is an improved version of RNN that learns effectively from long-term information. Each LSTM cell has a cell state (C̃t), a memory line that runs through the cell. A hidden state(ht) – output at each time step and three gates to control what to forget, what to remember, and what to output^44,45^. Table 2 presents the gates and states of the LSTM, along with their purpose and corresponding equations in detail. Where xt – input vector at time t, ht – hidden state at time step t, Ct – cell state at time step t, W and b are learnable weight matrices and biases, σ – sigmoid activation function, and tanh is the hyperbolic tangent function. Figure 2 shows the architecture of an LSTM.

**Table 2:** Details of the LSTM architecture.

Gate / State	Purpose	Equation	Activation
Forget Gate (f_t_)	Decides what information from the previous cell state to forget	f_t_ = σ(W_f_ · [ h_t−1_, X_t_ ] + b_f_)	Sigmoid
Input Gate (i_t_) & Candidate State (C̃_t_)	Decides what new information to add to the cell state.	i_t_ = σ(W_i_ · [ h_t−1_, X_t_ ] + b_i_) C̃_t_ = tanh(W_c_ · [ h_t−1_, X_t_ ]+b_c_)	Sigmoid
Output gate (O_t_) & Hidden State (h_t_)	Decides what to output from the current cell	O_t_ = σ(W_o_ · [ h_t−1_, X_t_] + b_o_) h_t_ = O_t_ * tanh(C_t_)	Sigmoid
Cell State Update (C_t_)	Combines previous state and new candidate information	C_t_ = f_t_ * C_t−1_ + i_t_ * C̃_t_ )	Tanh

### BiLSTM

**Fig. 3:**
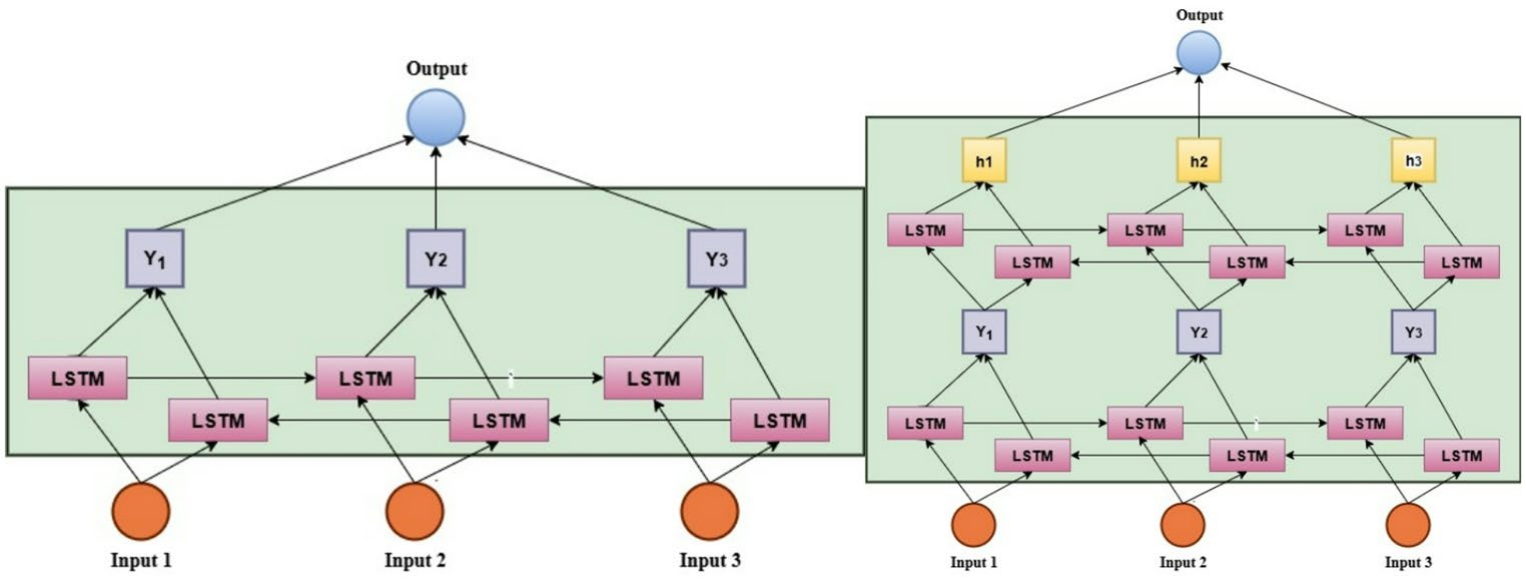
BiLSTM and stacked BiLSTM architecture.

An improved version of the traditional LSTM model, the Bidirectional Long Short-Term Memory (BiLSTM), is crafted to handle sequential data in both forward and reverse directions. The bidirectional processing increases the model’s capability for recognizing long-range dependencies and makes it especially effective for predicting time-series data^46^. In electric vehicle (EV) applications, BiLSTM is extensively used for deep learning tasks like state estimation, leading to enhanced accuracy and robustness. A stacked bidirectional LSTM model has several bidirectional LSTM layers stacked on top of one another^47^. Each layer takes input from the preceding layer, processes it in both forward and backward directions, and then passes it to the subsequent layer. Figure 3 explains the architecture of BiLSTM and stacked BiLSTM.

### Attention mechanism

The Attention mechanism assigns importance scores to various time steps that the model concentrates on essential information selectively. Single-head attention mechanism means, the features of each time step contribute differently in the final prediction. The weight matrix is trained to identify which parts of the sequence are more important. The softmax function gives the probability distribution from the scores.

This approach allows the model to prioritize relevant information while filtering out extraneous features, thereby enhancing interpretability. Attention weights give insights into the model’s decision-making process by highlighting the most influential inputs. This improves the interpretability and computational efficiency^48^.

3 $$\:W\in\:{R}^{dx1}$$

In Eq. 3, W is a vector with d rows and 1 column, and each element of W is a real number.

4 $$\:s=xW$$

5 $$\:a=softmax\left(s\right)$$

6 $$\:c=\sum\:_{n=1}^{T}{a}_{n}{x}_{n}$$

In Eqs. 4,5, and 6, s is a vector of scores (importance of each time step), and c is the context vector summarising the sequence. $\:a$_n_ is the attention weight for time step n, and x is the feature vector.

### KANBiLSTMAtt

The amalgamation of the Kolmogorov-Arnold Network (KAN) with Bidirectional Long Short-Term Memory (BiLSTM) presents numerous benefits for nonlinear battery behaviour, especially in SoC estimation. KAN accurately approximates intricate, high-dimensional functions without the need for conventional activation functions, facilitating enhanced feature representation and dimensionality reduction prior to sequential processing. KAN approximates high-dimensional functions using spline-based or activation-free transformations. KAN extracts non-linear relationships efficiently before passing structured representations to BiLSTM.

By elucidating complex nonlinear dependencies in battery dynamics, KAN augments the feature space, enabling the BiLSTM to concentrate on extracting temporal correlations with more efficiency. The bidirectional characteristic of BiLSTM enhances the predictive model by acquiring dependencies from both preceding and subsequent states, leading to a more precise and resilient SoC estimate. To improve generalization and lead to expedited convergence, KAN provides a systematic transformation to reduce the complex BiLSTM architectures, and it focuses only on temporal dependencies. The integration of an Attention mechanism markedly enhances the model’s interpretability and efficiency by dynamically assigning significance to various time steps, enabling it to concentrate on the most vital information under diverse settings. The Attention method enhances resilience by emphasizing critical features and reducing sensitivity to noise compared to traditional fully linked layers. Table 3 describes the comparison of the different deep learning models with the proposed model. Furthermore, it enhances computational performance by selectively analysing pertinent data, hence minimizing redundant calculations. This hybrid approach optimizes predictive accuracy, improves computing speed, and facilitates model generalization, rendering it extremely suitable for real-time battery state estimation.

**Table 3:** Comparison of deep learning models.

Model	Gradient stability	Memory ability	Ability to model nonlinearity	Interpretability	Use in sequence tasks
RNN	No	Yes	No	No	Yes
LSTM	Good	Good	Moderate	Low	Good
GRU	Better than LSTM	Good	Moderate	Low	Good
BiLSTM	No	Better than LSTM, as it considers the past and future	Moderate	low	Excellent
KAN	No, due to functional mapping	High, it directly learns mappings	Very high	High (spline interpretable)	No
Attention mechanism	No, learns dynamic importance	Moderate, based on attention structure	Depends on the base model	High	Very good
Proposed model	Least gradient problem	Very high, as it combines functional mapping and bidirectional context	Best	High	Best

## Optimization algorithms used in the proposed model

The selection of hyperparameters, which encompasses the number of hidden neurons, the number of hidden layers, the learning rate, and the activation function, exerts a considerable impact on the efficacy model. An increase in the number of hyperparameters leads to a geometric escalation of the search space. Moreover, the interdependence of each hyperparameter implies that modifications to these parameters can yield suboptimal results. Conventionally, methodologies such as grid search and random search are utilized for the optimization of hyperparameters. However, due to their substantial spatial demands, the considerable time required for training an individual model, and their high computational costs, these methods are not extensively employed. A multi-objective optimization algorithm is used to resolve issues where two or more objectives clash. Because of its effectiveness, diversity preservation, and capacity. Hence the proposed model uses Optuna for hyperparameter tunning and NSGA-II to optimize the training time, loss and computation cost. Hyperparameters are categorized into model and optimizer hyperparameters. Model hyperparameter is number of neurons in the network and optimizer parameters are batch size, learning rate, and epoch. Choosing the right hyperparameters plays a key role in improving model training efficiency and minimizing training error. Based on insights from previous studies, we define the search range for key hyperparameters: neuron sizes of [16, 32, 64, 128, 256], batch sizes of [16, 32, 64, 128, 256], and training epochs of [20, 50, 100, 150, 200]. In addition, we use a dynamic tuning approach to optimize the learning rate, which helps boost both the model’s performance and its convergence speed. The best values for neuron size, batch size, and number of epochs are identified using optuna.

### Optuna

Hyperparameter tuning plays a crucial role in model generalization and the overall performance of the machine learning model. Optuna is an automated hyperparameter optimization method that effectively identifies the optimal parameters for a machine learning model^49^. The process involves establishing an objective function that encompasses the training and assessment of a model, yielding a performance metric such as loss or accuracy. Optuna thereafter performs numerous trials, each embodying a distinct configuration of hyperparameters, to optimize the specified measure. Throughout these trials, it employs sophisticated sampling techniques such as the Tree-structured Parzen Estimator (TPE) to recommend advantageous hyperparameter choices informed by historical performance. Moreover, Optuna facilitates early termination via pruning, which suspends unpromising trials to conserve time and resources. The framework is adaptable and interoperable with any machine learning library, additionally facilitating multi-objective optimization when many metrics must be evaluated. Upon optimization, the optimal hyperparameters can be readily extracted and implemented in the final model.

The objective function is a core component used by Optuna to define the search space and evaluate different hyperparameter combinations during the optimization process. In this function, various hyperparameters are suggested dynamically for each trial. Specifically, the optimizer name is selected from a categorical choice among commonly used optimizers like Adam, SGD, and RMSprop. The learning rate is explored using a logarithmic uniform distribution, allowing the algorithm to search across several magnitudes (from 1e-4 to 1e-2). The hidden dimension, representing the number of neurons in the hidden layers, is selected from an integer range between 16 and 128. Similarly, the batch size, which affects training efficiency and convergence, is chosen from a set of common values: 16, 32, and 64. Two additional parameters, kan units (number of units in a Kolmogorov-Arnold Network layer) and number of knots (related to spline interpolation complexity), are also tuned—kan units is chosen in steps of 16 from 16 to 64, while number of knots is selected from a range of 3 to 10. These suggestions collectively allow Optuna to intelligently explore and identify the most effective hyperparameter configuration for optimizing the performance of the machine learning model.

### Non-dominated sorting genetic algorithm (NSGA)

Fine-tuning the model hyperparameters is essential for obtaining the optimal model performance, which is based on accuracy, computational efficiency, computational time, model size, and other factors. The NSGA is a popular evolutionary method for multi-objective optimization used to find a set of optimal solutions when there are two or more conflicting objectives^50^. In contrast to conventional optimization methods that prioritize a one optimal solution, NSGA is engineered to identify a broad array of optimal solutions, hence creating a Pareto front. This renders it especially advantageous for issues with contradictory objectives, where enhancing one purpose may compromise another. NSGA employs a non-dominated sorting algorithm to assess and rank solutions. A solution is deemed non-dominated if no other solution exceeds it in all objectives. Higher-ranked solutions form the Pareto front and are prioritized during the selection process. To attain a well-distributed array of solutions along the Pareto front, NSGA utilizes crowding distance, which measures the solution density around a certain point. Sorts the population into different Pareto fronts. The first front contains the best solutions, and subsequent fronts contain solutions dominated by those in earlier fronts. Crowding distance is used to maintain diversity in the population. It measures how close a solution is to its neighbours in the objective space, and it chooses the well-distributed solutions across the Pareto front. Combines parents and offspring into a single population and selects the best individuals from this combined pool to pass on to the next generation. So, no good solution is lost; this improves convergence and stability. This facilitates the maintenance of solution diversity and prevents early convergence.

The NSGA follows the concepts of genetic algorithms, utilizing selection, crossover, and mutation operators to progress the population throughout subsequent generations. The amalgamation of elitism and genetic mechanisms enables efficient navigation of the solution landscape. Within the objective function, the model is compiled using the selected optimizer (e.g., Adam, SGD, or RMSprop) with Mean Squared Error (MSE) as the loss function, which is suitable for regression tasks such as SoC estimation. After training, a small batch of input data is passed through the model to evaluate its inference performance, and the time taken for this prediction is measured as inference time, which reflects how fast the model responds during deployment. To encourage model simplicity and interpretability, an additional metric called interpretability is introduced, calculated as the inverse of the hidden dimension (1 / hidden dimension). This assumes that models with fewer neurons are easier to interpret and debug. Finally, the objective function returns multiple metrics—training loss (loss train), training duration (training time), model complexity (model size), inference speed (inference time), and interpretability enabling multi-objective optimization using NSGA-II or similar algorithms. This approach allows the search to consider not only accuracy but also computational efficiency and model simplicity.

**Fig. 4:**
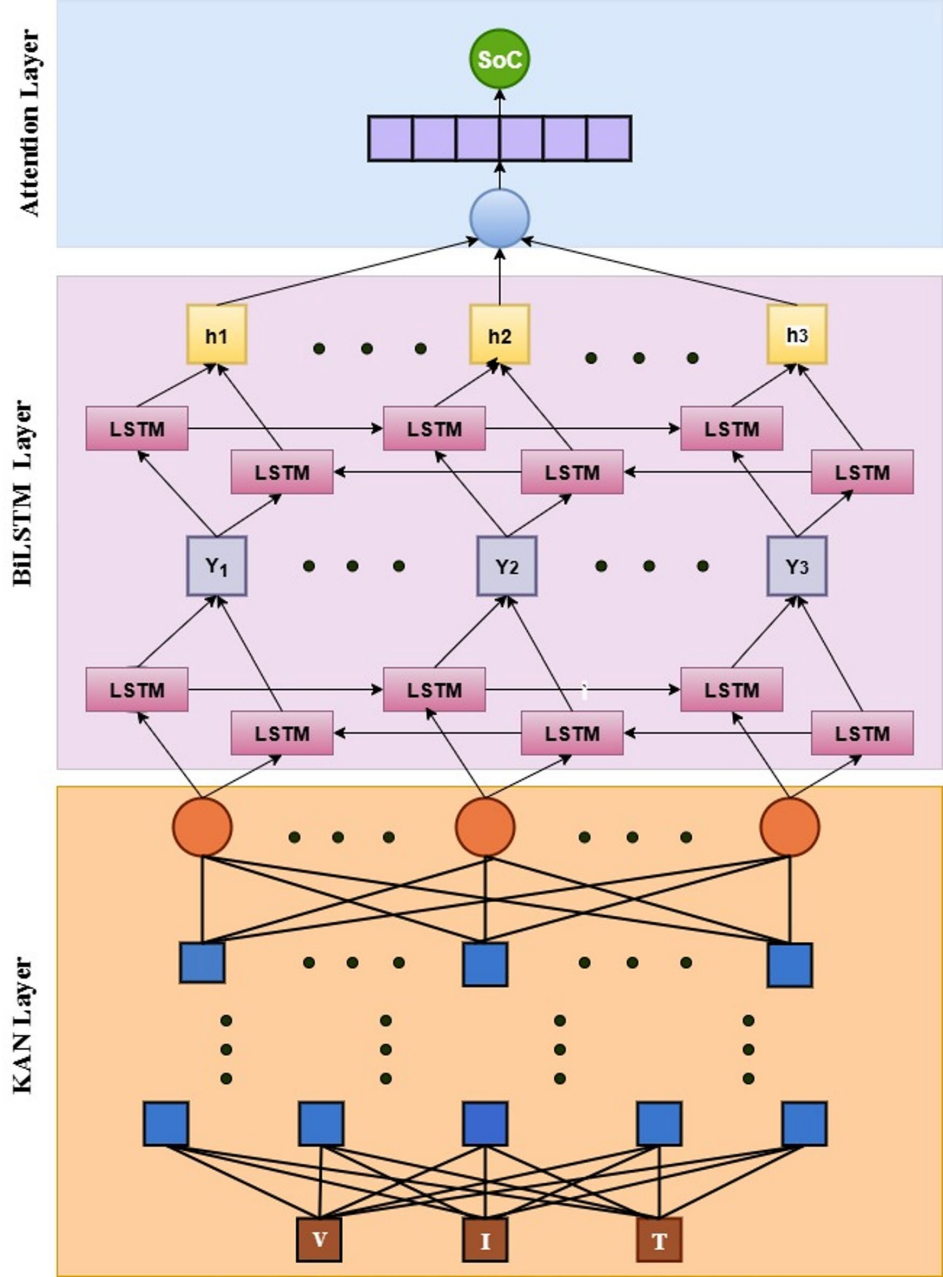
Structure of the proposed model.

Figure 4 shows the structure of the proposed hybrid deep learning model. In this model, three layers are used. They are KAN, a stacked BiLSTM, and followed by an Attention layer. The features are voltage, current, and temperature, are given to the input of the KAN layer. This model integrates KAN using a spline-based activation function, a bidirectional LSTM, and an attention mechanism to predict a sequence of SoC at all timesteps. The KAN layer applies a linear transformation to the input, followed by a piecewise cubic spline function, where the positions of spline knots are trainable. This non-linear transformation captures complex relationships in the input data. The transformed output is then fed into a stacked BiLSTM to learn temporal dependencies from both past and future directions. After temporal encoding, an attention layer computes weighted importance scores for each timestep, allowing the model to focus on the most relevant time features. The resulting context vector is passed through a fully connected dense layer to produce SoC predictions for each timestep.

**Fig. 5:**
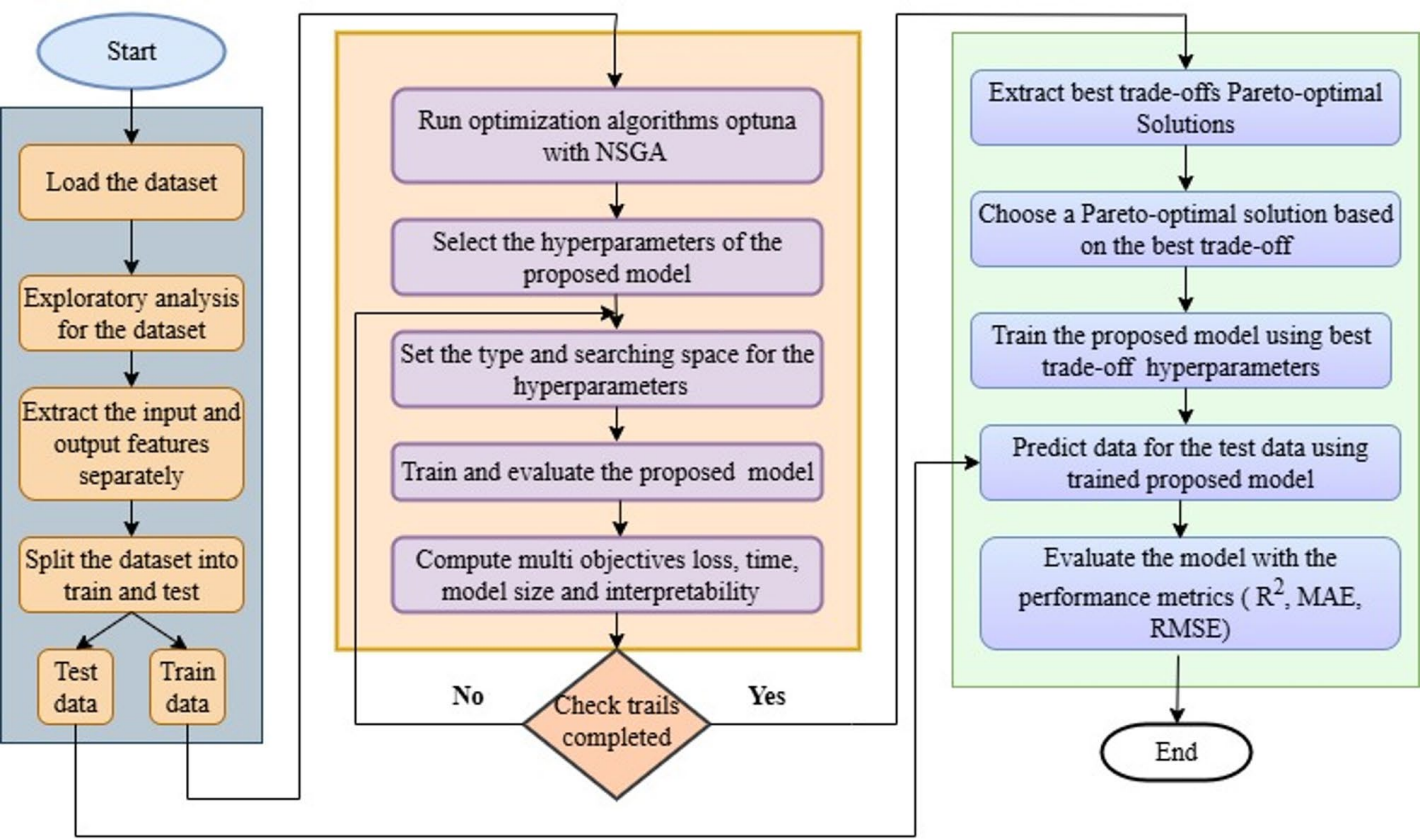
Flowchart for the proposed model.

Figure 5 shows the flowchart for the proposed method. The algorithm starts with dataset loading, preprocessing, extracting the voltage, current, and temperature are input features, and SoC as an output feature. Splitting the dataset into train and test. Hyperparameter batch size is used for how much data is fed into the model at each time step, epoch for how many numbers of model iterations, and learning rate is the model weight update rate. Run the optimization algorithms optuna with NSGA for the training dataset. Train and evaluate the proposed model, including computational cost, for a specified number of trials. Extract the best trade-off Pareto solutions, and train the model with those solutions. Evaluate the model with the test data.

## Dataset description

In these experiments, two different datasets are used from the open-source datasets for battery experimental data described in Table 4.

**Table 4:** Dataset Description.

Parameter	Dataset 1	Dataset 2
Brand	Samsung	LG
Type	INR 18,650 -20R (LiNiMnCoO2)	INR 18,650 HG2 (LiNiMnCoO2)
Nominal capacity	2000mAh	3000mAh
Nominal voltage	3.6 V	3.6 V
Maximum charging voltage	4.2 V	4.2 V
Minimum discharging voltage	2.5 V	2.5 V

### Dataset 1

The data are obtained from Hamilton’s McMaster University and the University of Maryland CALCE centre. The INR18650-20R battery is manufactured by Samsung, is tested in the Arbin BT200 test system^51^. Terminal voltage, current, and sampling time are the features of the dataset. The initial capacity is the initial SoC of the battery; for this experiment, it is set to 80%. Using the Coulomb counting method, the SoC is calculated. The dataset includes a thorough testing regimen aimed at assessing battery performance and enabling precise SoC calculations under various scenarios. The profiles include the driving cycles such as, Dynamic Stress Test (DST), the Federal Urban Driving Schedule (FUDS), the US06 Highway Driving Schedule, and the Beijing Dynamic Stress Test (BJDST). These profiles cover a wide range of real-world driving conditions, including frequent acceleration and deceleration (DST), urban stop-and-go traffic (FUDS), high-speed highway scenarios (US06), and mixed urban patterns typical of Beijing traffic (BJDST). This ensures comprehensive validation of the model’s performance under diverse operating conditions.

### Dataset 2

A brand new LG HG2 cell nominal capacity of 3Ah was tested using a 75-amp, 5-volt Digatron firing circuits universal battery tester channel. 0.1% full scale of accuracy for voltage and current measurements. Three experimental data points are obtained for three different temperatures, 0 °C, 10’C and 25 °C. The dataset, comprising various drive cycles recorded at different temperatures within a single file, was first loaded for processing. Each dataset was resampled at 1 Hz to maintain consistency and ensure uniform temporal resolution. The testing commenced with the characterization of Open Circuit Voltage (OCV), executed by low-current and incremental-current methodologies at a temperature of 45 °C^52^. These assessments enable the development of a reliable OCV-SoC relationship, which is critical for model-based SoC estimation methodologies. In conjunction with OCV evaluations, dynamic load profiles were employed to simulate real-world operational scenarios. The resampled datasets were then synchronously concatenated, allowing for a comparative analysis of ampere-hour (Ah) consumption across different temperatures. The complete dataset was consolidated for voltage, current, temperature, and SoC. Data cleaning was performed to eliminate anomalies such as voltage, current, and Ah spikes, ensuring the integrity of the dataset.

In data preprocessing, normalization is used to generalize the features. The voltage, current, and temperature in the dataset are different in magnitude. Min-Max normalization is a method to map the values in the 0–1 range.

7 $$\:\widehat{{X}_{i}}=\:\frac{{X}_{i}-\:{X}_{min}\:}{{X}_{max}-\:{X}_{min}}$$

In Eq. 7, X is the input data, X_i_ is current input, X_min_ is the minimum, X_max_ is the maximum, and X̂_i_ is the mapped data (0–1) obtained from normalization.

## SoC estimation performance criteria

To evaluate the performance accuracy of different machine learning models in SoC estimation, namely mean absolute error (MAE), root mean square error (RMSE), and coefficient of determination (R^2^).

8 $$\:MAE=\frac{1}{{N}_{m}}{\sum\:}_{k=1}^{{N}_{m}}\left|{SoC}_{k}-{pSoC}_{k}\right|$$

9 $$\:RMSE=\sqrt{\frac{1}{{N}_{m}}{\sum\:}_{k=1}^{{N}_{m}}{\left({SoC}_{k}-{pSoC}_{k}\right)}^{2}}$$

10 $$\:{R}^{2}=1-\frac{{\sum\:}_{k=1}^{{N}_{m}}{\left({SoC}_{k}-{pSoC}_{k}\right)}^{2}}{{\sum\:}_{k=1}^{{N}_{m}}{\left({SoC}_{k}-{mSoC}_{k}\right)}^{2}}$$

In Eqs. 8,9,10, SoC is the actual value of SoC, pSoC is the predicted value of SoC, and N_m_ is the number of inputs.

## Input features of the model

**Fig. 6:**
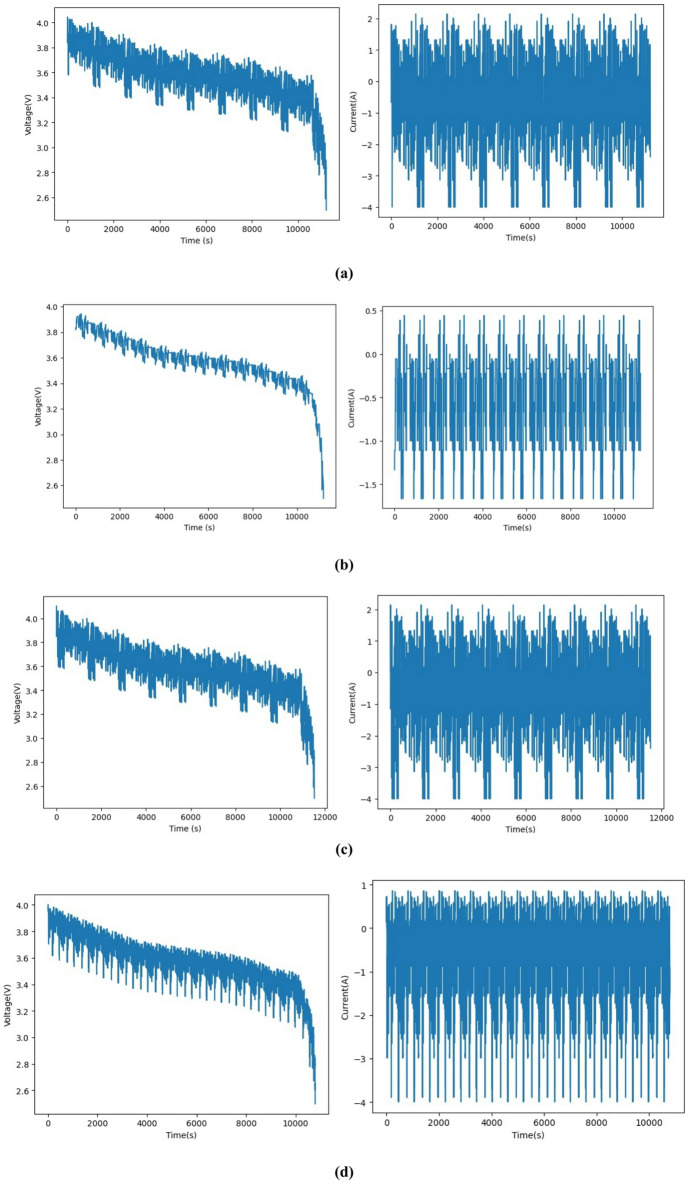
Voltage and current profiles for dataset 1: (**a**) DST, (**b**) BJDST, (**c**) FUDS, and (**d**) US06.

**Fig. 7:**
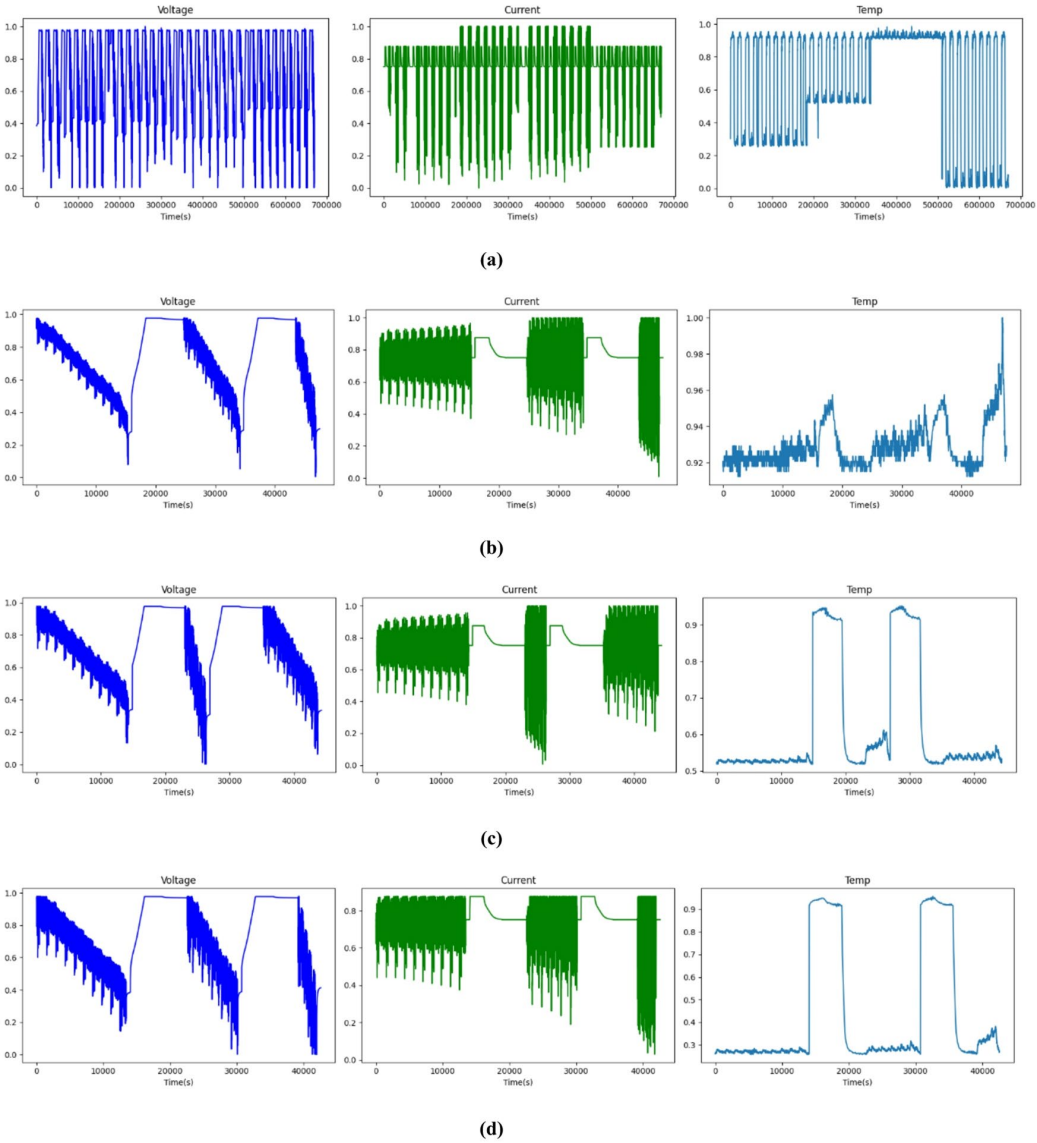
Voltage, current, and temperature profiles of dataset2 in different temperatures. (**a**) Combined, (**b**) 25 °C, (**c**) 10 °C, and (**d**) 0 °C.

Figure 6 shows the voltage and current profiles for four driving cycles for dataset (1) Here, the temperature is constant at 45 °C. It clearly shows that the voltage and current vary depending on the driving cycles of the vehicle. Figure 7 shows the voltage, current, and temperature profiles of dataset (2) Here, the temperatures are 25 °C, 10 °C, and 0 °C. These three variables are the input to the proposed model.

## Results and discussion

The proposed model was applied to the two datasets to estimate the SoC. All experiments were conducted using Google Colab, which provides a cloud-based environment with access to an NVIDIA GPU, along with a local system running Windows OS equipped with 32 GB of RAM and a 3.19 GHz Intel Core i7 CPU for additional processing and analysis.

### Dataset-1

**Fig. 8:**
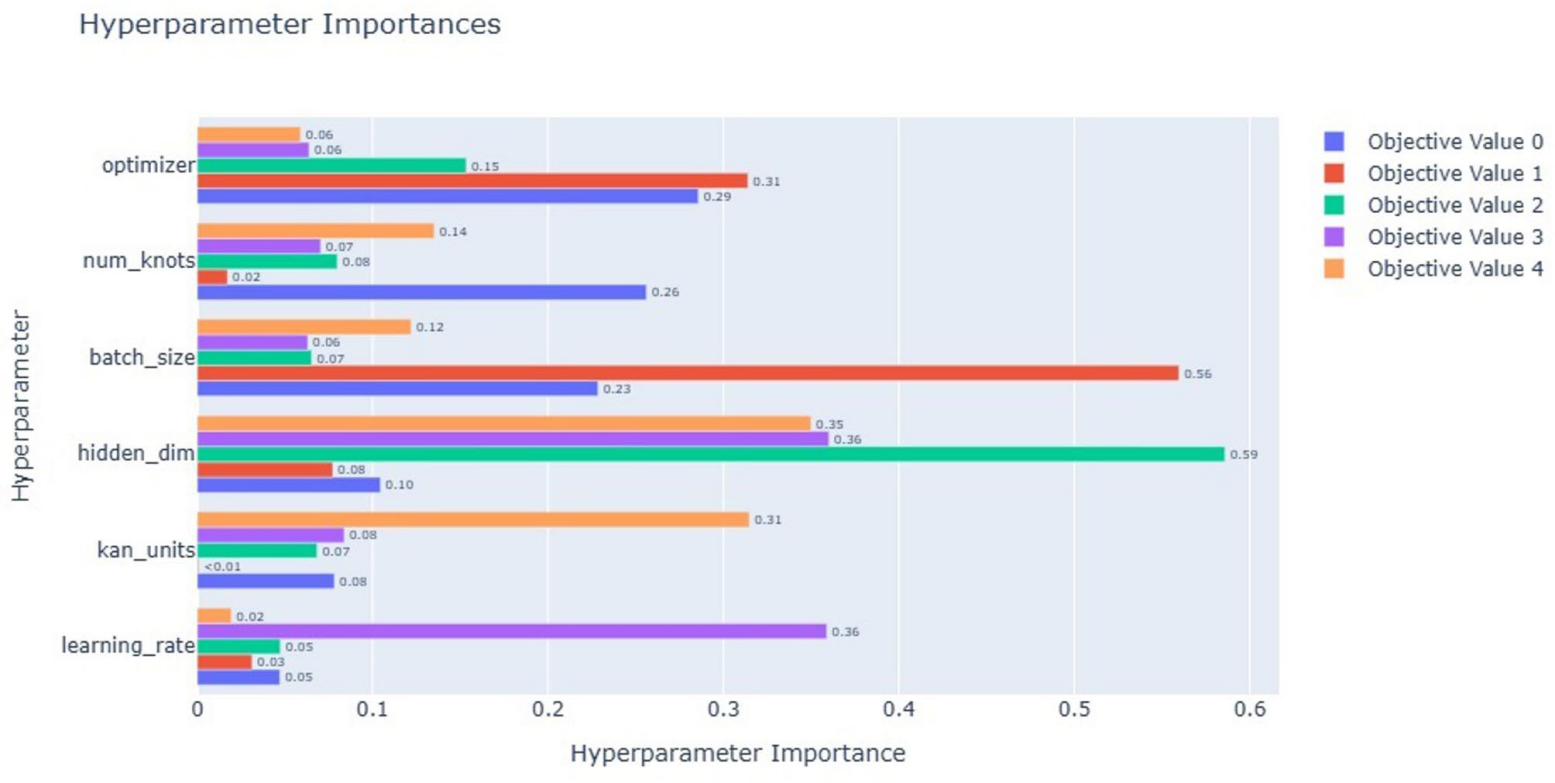
Hyperparameter importance for the objectives of dataset1: Objective value 0- model size, 1- model loss, 2- train time, 3-inference time, 4-interpretability.

**Fig. 9:**
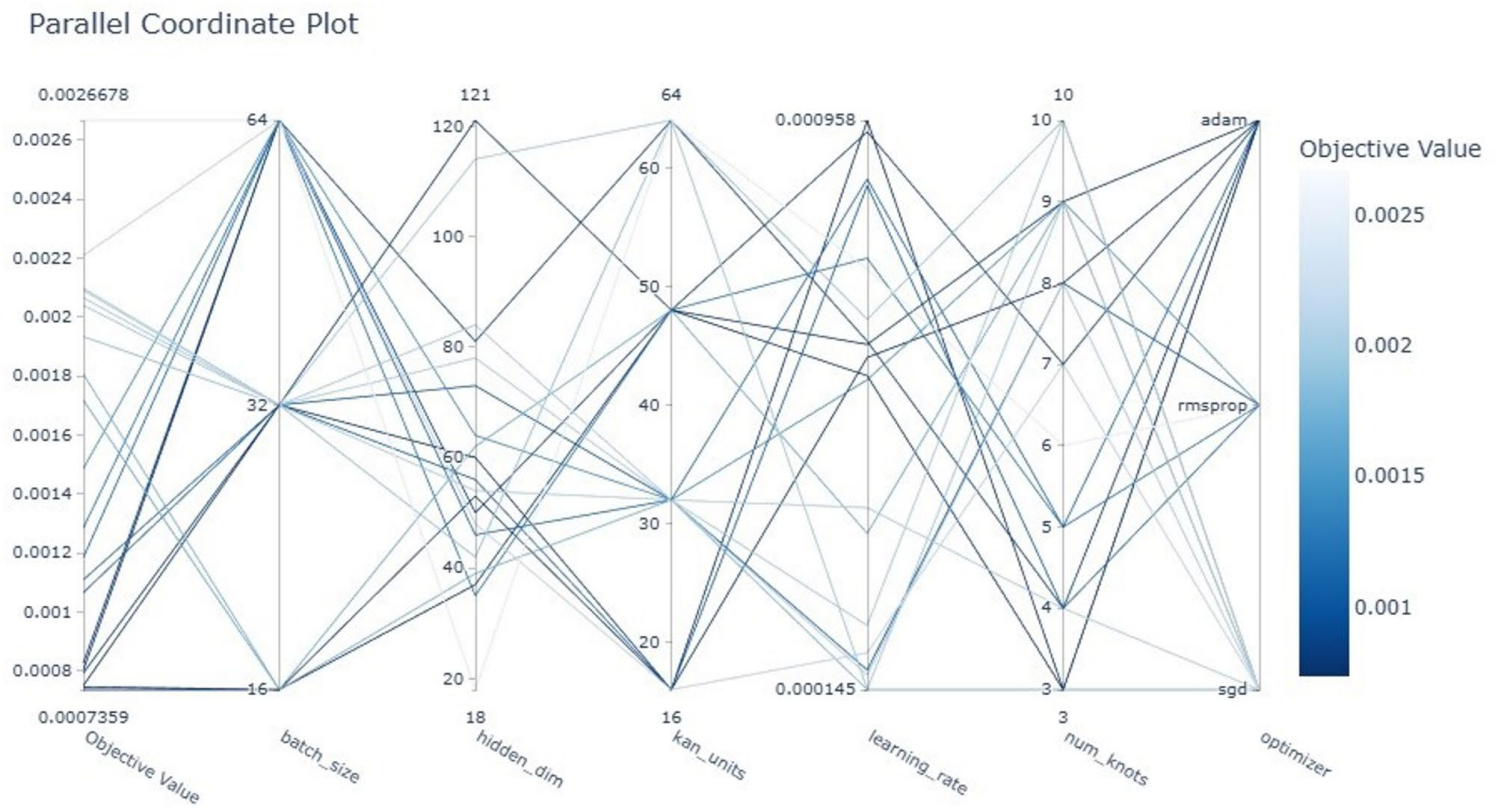
Parallel coordinate plot for the hyperparameter of dataset1.

In this paper, the DST at 25 °C dataset from CALCE is used to train the proposed model, and other datasets, BJDST, FUDS, and US06, are used to test the model. The optimizer optuna is used to tune the hyperparameters of the model, and NSGA is used for multi-objective optimization. Hyperparameters are the number of kan units, hidden layers, number of kan knots for the spline, batch size, learning rate, and optimizer. The objectives are training error, time, model size, inference time, and interpretability. Figure 8 shows the hyperparameter importance for each objective. Hidden dimensions are of more importance for the objectives model size, inference time, and interpretability. Learning rate is important for inference time, kan units, and the number of kan knots are important for interpretability and loss. Batch size is important to minimize time and loss. The optimizer is used to minimize training time and loss. Figure 9 shows the parallel coordinate plot for the hyperparameter of dataset1, which is used as a visualization tool to analyse multidimensional data, especially in hyperparameter tuning.

**Fig. 10:**
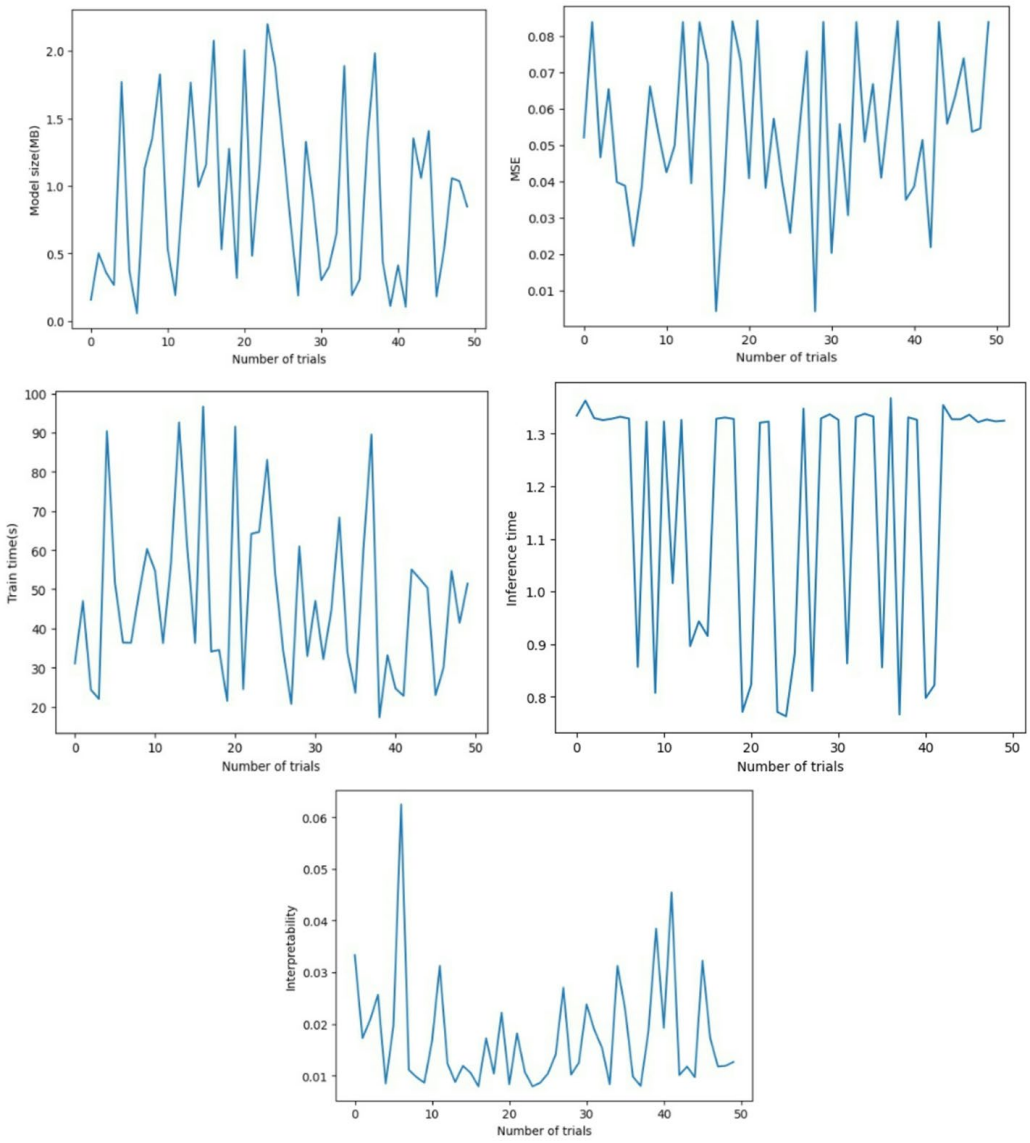
Plot for trial vs. objectives.

**Fig. 11:**
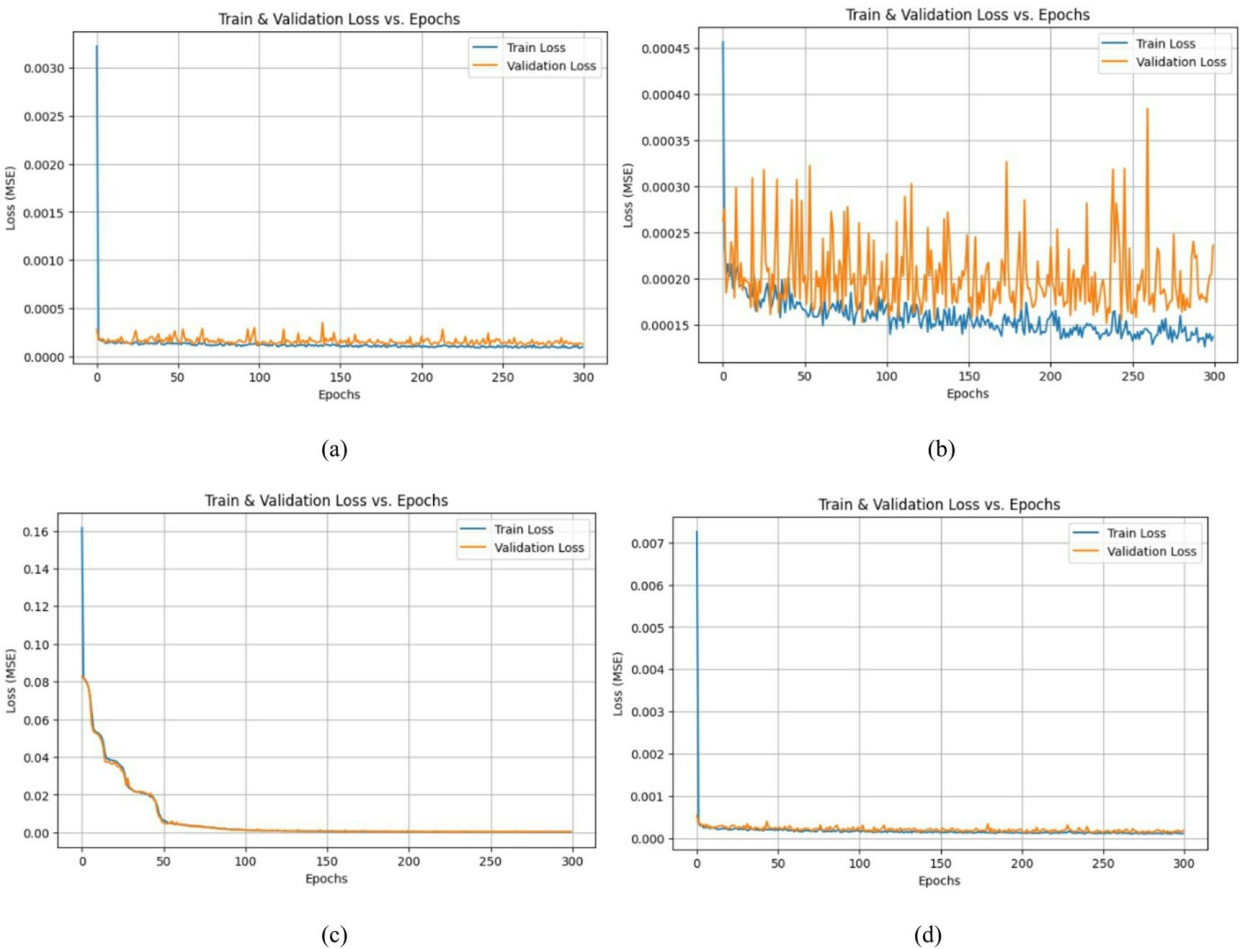
Training and validation loss for driving cycles (**a**) DST, (**b**) FUDS, (**c**) US06, and (**d**) BJDST.

An Optuna study is created with multi-objective optimization using the NSGAII Sampler, which implements the Non-dominated Sorting Genetic Algorithm II (NSGA-II). This evolutionary algorithm is well-suited for optimizing multiple conflicting objectives simultaneously. The directions parameter specifies the goals for each objective returned by the objective function: four of them—training loss, training time, model size, and inference time—are to be minimized, while interpretability is to be maximized. Optuna executes 50 different trials, each with a different combination of hyperparameters suggested by the NSGA-II sampler. This process searches for a Pareto-optimal set of solutions, where improvements in one objective cannot be made without compromising another. As a result, it helps identify models that strike the best trade-offs among accuracy, efficiency, and simplicity. Figure 10 demonstrates high efficiency and accuracy, with a compact size of less than 2 MB, making it well-suited for resource-constrained environments. It achieves a low Mean Squared Error (MSE) of less than 0.08, indicating strong predictive performance. The model also exhibits fast training, with a total training time under 90 s, and a low inference time of approximately 1.3 s, ensuring real-time applicability in practical battery management systems. From Table 5, train the proposed model with the best hyperparameter values are batch size 64, number of neurons in the hidden layer 55, kan units 16, learning rate 0.000315, number of kan knots for the spline 3, and the optimizer is Adam. Adam optimizer is used to optimize the weights for every time step. It enables faster convergence compared to the random gradient. Figure 11 shows the training and validation loss for the different cycles.

**Table 5:** Hyperparameters for dataset1.

Parameter	Value
Hidden dimension	55
Kan units	16
Number of knots	3
Batch size	64
Learning rate	0.000315
Optimizer	Adam
Loss function	MSE

**Fig. 12:**
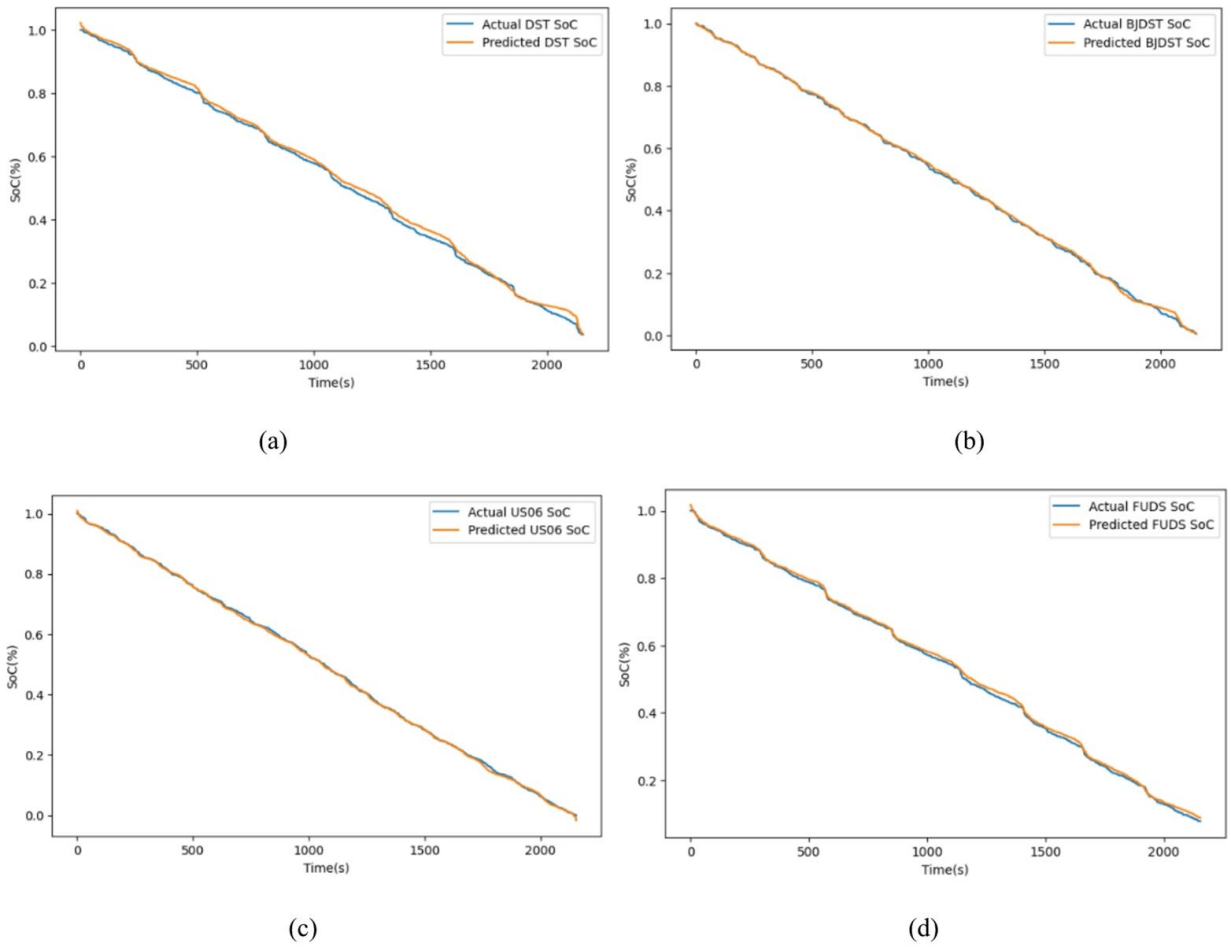
Actual vs. predicted SoC for the driving cycle (**a**) DST, (**b**) BJDST, (**c**) US06, and (**d**) FUDS.

**Fig. 13:**
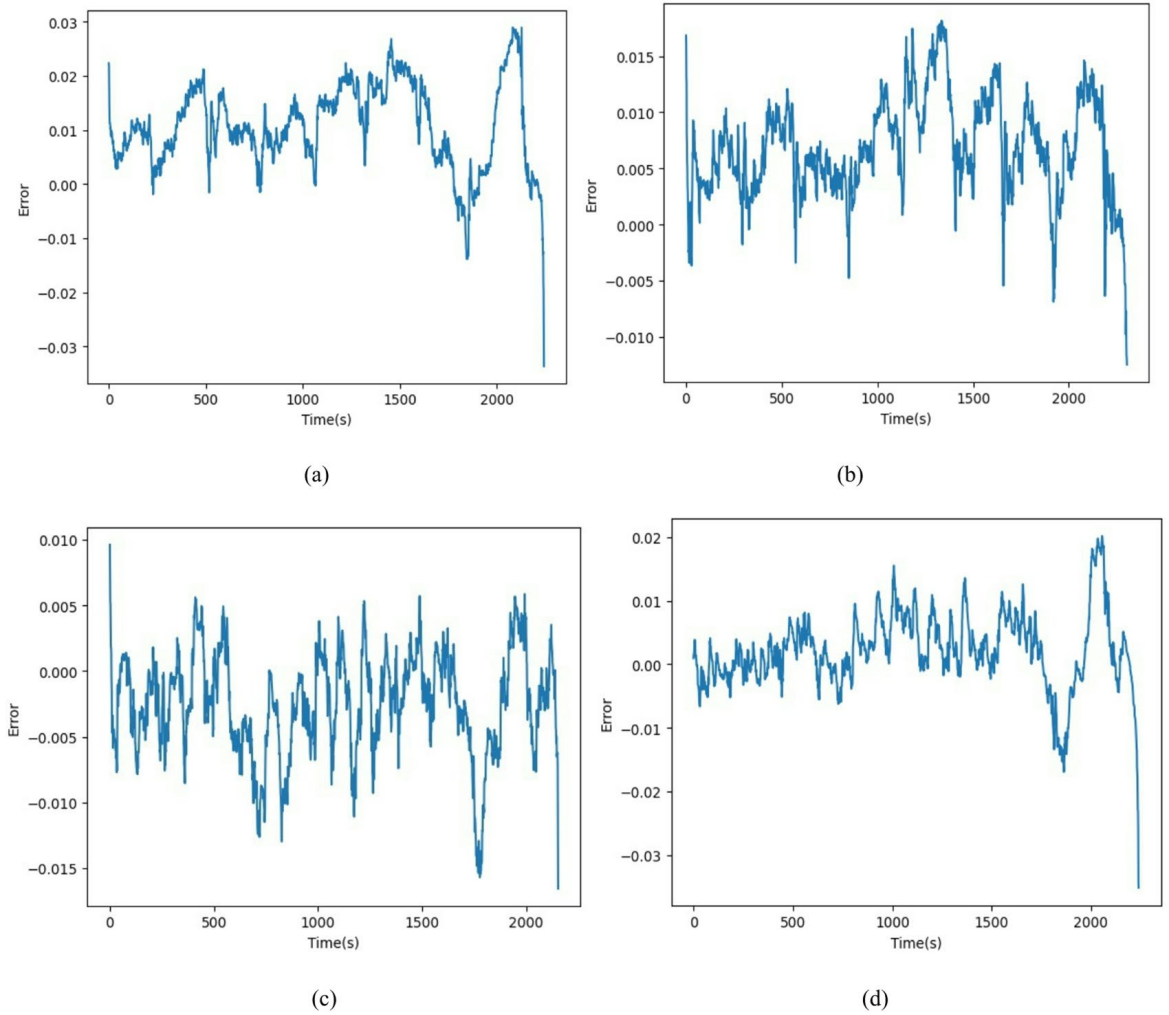
Error for the driving cycle (**a**) DST, (**b**) FUDS, (**c**) US06, and (**d**) BJDST.

Figure 12 visually compares the actual State of Charge values with the predicted SoC values for four different standard driving cycles. For DST, the predicted SoC closely follows the actual trend, though minor deviations may appear during rapid current transitions. This reflects the model’s ability to handle dynamic stress, but with slightly higher error due to aggressive patterns for BJDST, predicted SoC almost perfectly overlaps with the actual SoC. This demonstrates high accuracy in urban traffic scenarios with less fluctuation. For US06, even under aggressive high-speed driving, the model tracks the SoC very precisely. This highlights its robustness under fast and frequent changes in driving conditions. For FUDS, the model performs well in simulating urban stop-and-go traffic, with good alignment between predicted and actual SoC curves. Figure 13 validates the model’s ability to keep prediction errors minimal across various real-world driving scenarios. Overall that the proposed model generalizes well for all four driving scenarios.

**Table 6:** Performance measurement of SoC Estimation for dataset 1.

	DST	BJDST	FUDS	US06
RMSE	0.0232	0.0135	0.0158	0.0108
MAE	0.0181	0.0104	0.0123	0.0082
R^2^	0.9937	0.9980	0.9970	0.9986

Table 6 presents the performance metrics of the proposed model evaluated under four standard driving cycles: DST, BJDST, FUDS, and US06. Each of these driving cycles represents different real-world driving conditions such as acceleration, deceleration, speed variation, and load patterns. The evaluation metrics include measuring the standard deviation of prediction errors, capturing the average magnitude of the errors, and determining how well the model explains the variability of the target. For all the driving cycles, the RMSE is less than 0.02, the MAE is around 0.01, and the R^2^ is 99%. The proposed model yields better performance for SoC estimation.

### Dataset − 2

**Fig. 14:**
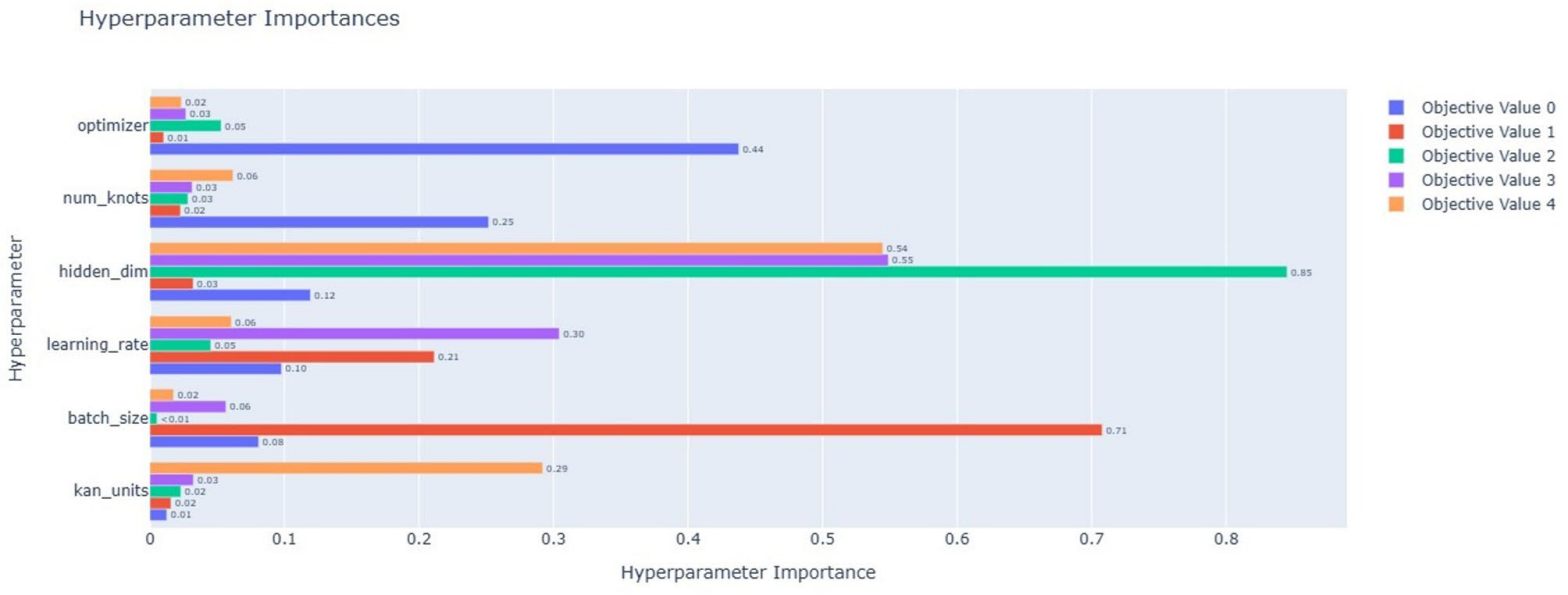
Hyperparameter importance for the objectives of dataset2: Objective value 0- model size, 1- model loss, 2- train time, 3- inference time, 4-interpretability.

Voltage, current, and temperature are the inputs, and the SoC is the output of the dataset that is used to train the model. And the model is tested for 25 °C, 10 °C, and 0 °C separately. The optimizer optuna is used to tune the hyperparameters of the model, and NSGA is used for multi-objective optimization. Hyperparameters are the number of kan units, hidden layers, number of kan knots for the spline, batch size, learning rate, and optimizer. Figure 14 shows the hyperparameter importance for each objective. The objectives are training time, error, model size, inference time for a single forward pass, and interpretability.

**Fig. 15:**
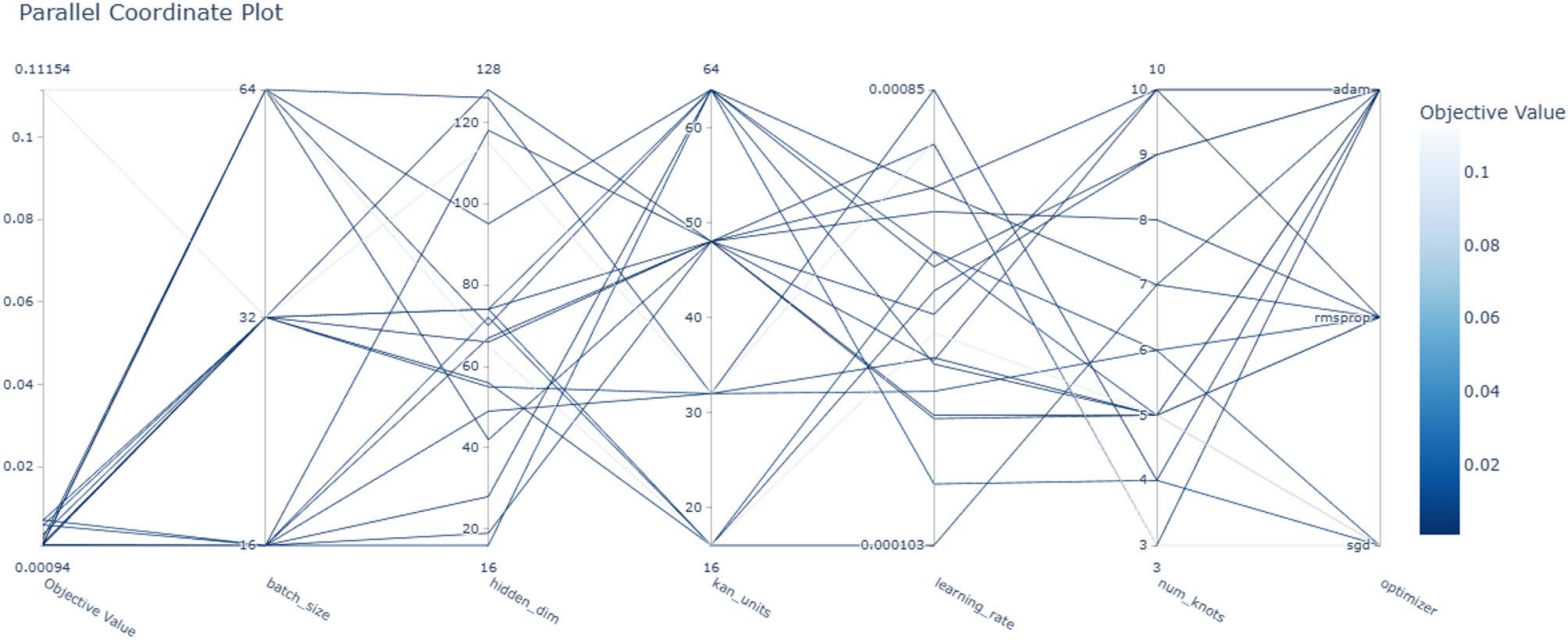
Parallel coordinate plot for the hyperparameter of dataset 2.

Figure 15 shows the parallel coordinate plot for the hyperparameter of dataset2. According to the trials evaluated by the optuna and NSGAII, the best trial had hyperparameter values are Hidden neurons 54, kan units 32, number of knots for the spline 5, batch size 32, learning rate 0.0005, and the optimizer is Adam, as shown in Table 7.

**Table 7:** Hyperparameters for the LG dataset.

Parameter	Value
Hidden dimension	54
Number of Kan units	32
Number of knots	5
Batch size	32
Learning rate	0.0005
Optimizer	Adam

**Fig. 16:**
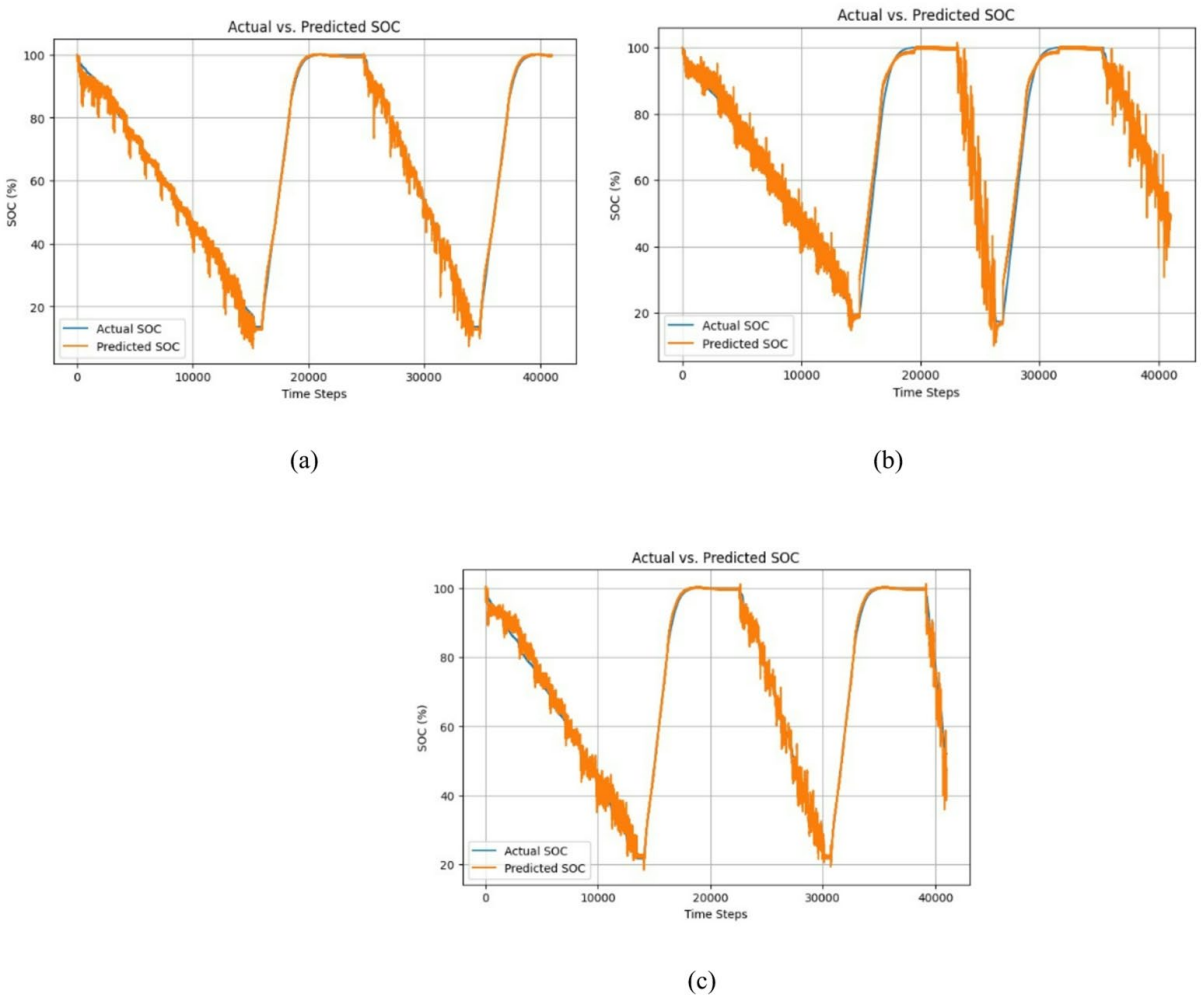
Actual vs predicted SoC for temperature (**a**) 25 °C, (**b**) 10 °C, and (**c**) 0 °C.

**Fig. 17:**
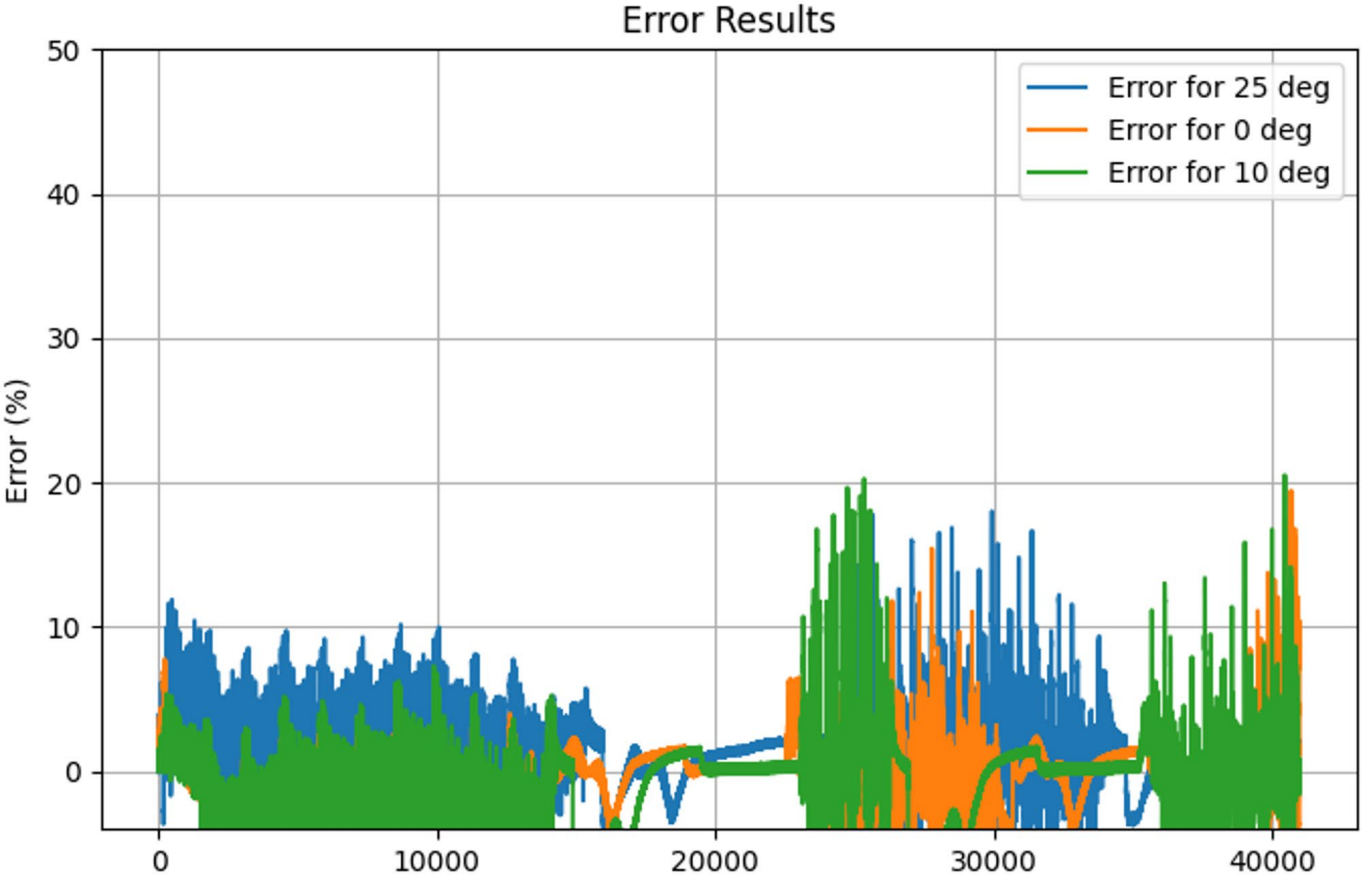
Error (%) for the temperature 25 °C, 10 °C, and 0 °C.

Figure 16 shows the Actual Vs Predicted SoC for the LG dataset at temperatures 25 °C, 10 °C, and 0 °C. As can be seen from the actual and the predicted SoC is the same. Figure 17 shows the error for 25 °C,0 °C, and 10 °C, which is mostly less than 10%. The proposed hybrid model predicts the SoC accurately.

#### Moving average for sensitivity analysis

**Fig. 18:**
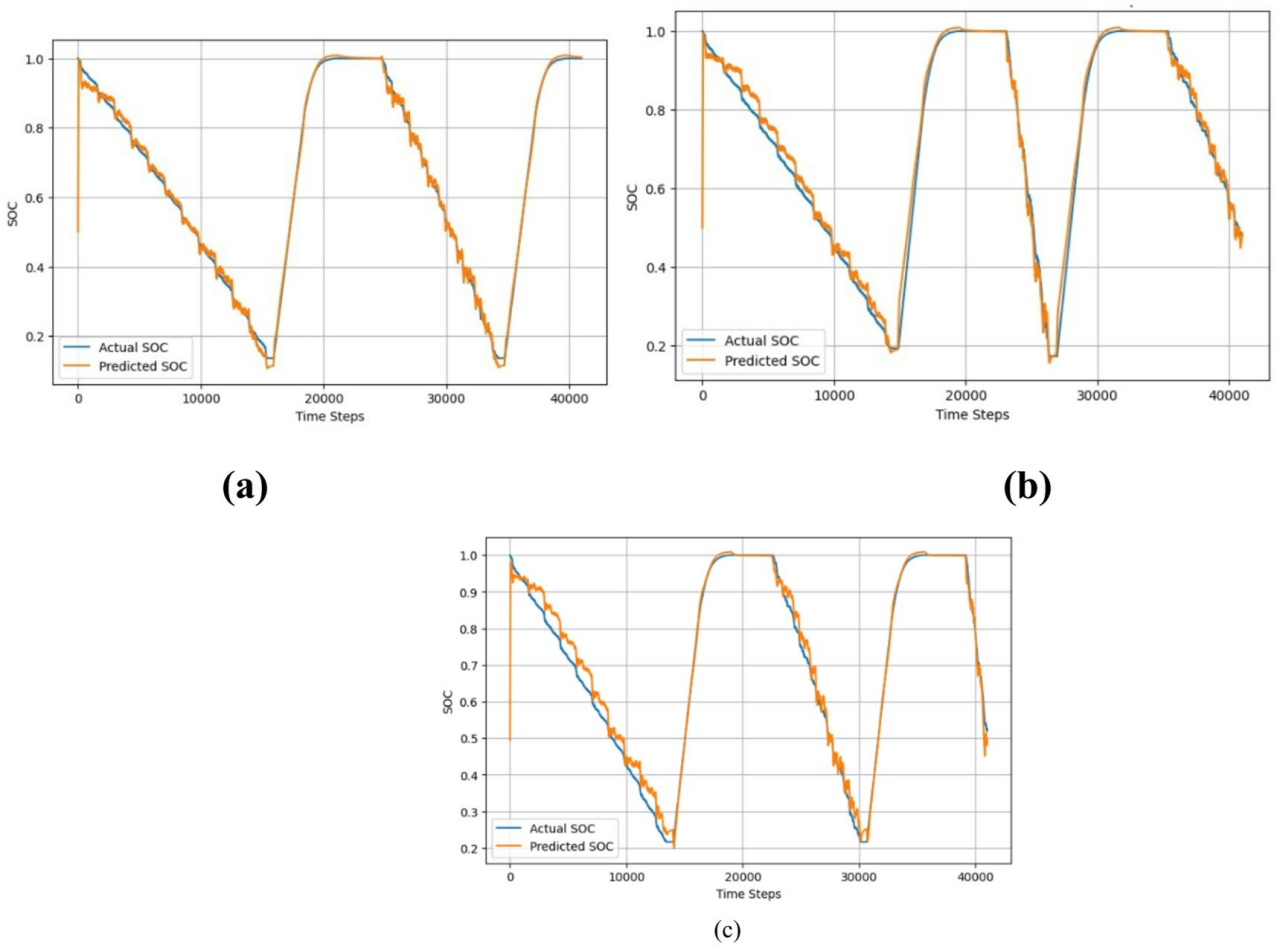
Actual Vs predicted SoC for a moving average window size of 100.

A moving average is a statistical technique used to smooth out short-term fluctuations and highlight longer-term trends or cycles in data. smoothing technique used to reduce noise in a dataset and highlight underlying trends. The data and a window size are the inputs, which define how many consecutive data points should be averaged together. A uniform filter generates equal weights that sum to one. This filter is then applied to the data. The result is a smoothed version of the original data where each value is the average of its neighbouring values, depending on the specified window size^53^. For this dataset, the Window size is 100. Figure 18 shows the actual and predicted SoC with a moving average window. This significantly reduces short-term fluctuations or noise in the data, making it easier to observe long-term trends or patterns. In equation SA_t_ – smoothed value at time x is the data, and N_m_ is the window size.

11 $$\:{SA}_{t}=\frac{1}{{N}_{m}}{\sum\:}_{i=1}^{{N}_{m}}{x}_{t-i}$$

**Table 8:** Performance measurement of SoC Estimation for the LG dataset.

	25 °C	10 °C	0 °C
RMSE	0.0180	0.0286	0.0306
MAE	0.0118	0.0198	0.0218
R^2^	0.9961	0.9891	0.9869

Table 8 shows the performance metrics for the LG dataset. for 25 °C, RMSE is 0.0180, MAE is 0.0118, and R2 is 0.9961. for 10 °C, and 0 °C, also it gives low RMSE and 99% as R^2^. This gives high accuracy. The proposed model predicts the SoC accurately 99.61%.

### Performance comparison analysis with the existing method

**Table 9:** Quantitative comparison with the existing papers.

Ref	Battery	Method	Temperature (°C)	Test case	Performance		
					MAE (%)	RMSE (%)	*R*^2^(%)
^54^	Samsung INR 18650-20R	NAG + BiGRU	45	FUDS	-	1.34	
^55^	Samsung INR 18650-20R	Model fusion with data-driven	45 45 45 45	DST BJDST FUDS US06	5.29 2.28 1.42 1.06	7.67 3.84 1.98 1.48	
^56^	Samsung INR 18650-20R	CNN-BiGRU-AUKF	45		2.30	2.80	
**Proposed**	**Samsung INR 18650-20R**	**KAN + BiLSTM + Att**	**45** **45** **45** **45**	**DST** **BJDST** **FUDS** **US06**	**1.81** **1.04** **1.23** **0.82**	**2.32** **1.35** **1.58** **1.08**	**99.37** **99.80** **99.70** **99.86**
^57^	LG 18,650 HG2	GRU-ATL	10 25 40	CC-CV	0.80 1.40 1.30	1.07 1.76 1.61	
^58^	LG 18,650 HG2	GA-GRU	20 30 45	BJDST DST US06	0.27 0.23 0.19	0.23 0.20 0.07	
**Proposed**	**LG 18,650 HG2**	**KAN + BiLSTM** ** + Att**	**0** **10** **25**	**CC-CV**	**0.02** **0.02** **0.01**	**0.03** **0.03** **0.02**	**98.69** **98.91** **99.61**

**Table 10:** Comparison with existing Algorithms.

Method	Key strengths	Key Limitations
Linear regression (LR)	Simple and easy to implement	Cannot capture nonlinear battery dynamics or temporal dependencies
Support vector regression (SVR)	Can model nonlinear relationships using kernels	Computationally expensive, sensitive to hyperparameters, and slower inference
LSTM	Good at capturing temporal dependencies	High computational cost, risk of overfitting, limited handling of nonlinear features
BiLSTM	Captures both past and future dependencies, improving temporal modelling	Increased complexity and computation compared to vanilla LSTM
GRU	Similar to LSTM but fewer parameters, faster training and inference	May not capture long-term dependencies as effectively as BiLSTM
**Proposed hybrid model**	**Integrates nonlinear feature extraction (KAN)**,** BiLSTM with attention**,** and dual-phase optimization for accurate**,** robust**,** and efficient SoC estimation**	**Requires offline training**,** but inference is fast and highly robust against noise**

Table 9 shows the quantitative comparison with the existing paper, and Table 10 highlights the key strengths and limitations of various SoC estimation methods. Linear Regression and SVR offer simplicity and nonlinear modeling capabilities, respectively, but struggle with complex temporal dynamics and require careful hyperparameter tuning. Standalone LSTM effectively models temporal dependencies but suffers from higher computational cost and overfitting risks. BiLSTM improves temporal feature extraction by considering both past and future states, while GRU offers faster computation with fewer parameters but slightly less ability to capture long-term dependencies compared to BiLSTM. In contrast, the proposed hybrid model combines nonlinear feature extraction (using KAN), BiLSTM with attention for enhanced temporal dependency modeling, and dual-phase optimization, resulting in accurate, robust, and computationally efficient SoC estimation suitable for onboard applications.

### Onboard computational efficiency and robustness evaluation

The proposed hybrid model is efficient for real-time onboard SoC estimation. During inference, it requires only straightforward calculations, enabling predictions to be generated within a few milliseconds per sample on standard hardware. To evaluate robustness, we tested the model by adding Gaussian noise to voltage and current inputs at different levels (e.g., 0.5%, 1%, 2%). The results show that the estimation accuracy (RMSE and MAE) degrades only slightly, demonstrating that the model remains accurate and stable even when sensor measurements are noisy, without adding extra computational cost during online use.

**Table 11:** Robustness evaluation results.

Noise level (% of std dev)	MAE (%)	RMSE (%)
0.0	1.81	2.32
0.01	1.92	2.42
0.03	1.98	2.59
0.05	2.35	2.98

Table 11 shows the MAE and RMSE of the proposed hybrid model evaluated on test dataset 1(CALCE -DST driving cycle) with different levels of Gaussian noise added to the voltage and current inputs. As the noise increases, the estimation errors increase only slightly, demonstrating the strong robustness of the model under measurement uncertainty.

### Adaptability of the proposed method to LiFePO₄ chemistry

To validate the generalizability and effectiveness of the proposed hybrid deep learning framework with the publicly available A123 LiFePO4 battery dataset from CALCE as the test case. The dataset provides voltage, current, and SoC measurements across different driving cycles.

**Table 12:** Performance of the proposed method with the LiFePO_4_ battery.

Test case	MAE (%)	RMSE (%)	*R* ^2^ (%)
DST	2.89	3.25	95.43
US06	2.04	2.35	94.56
FUDS	2.38	2.69	95.61

Table 12 demonstrates that the proposed model achieves accurate SoC estimation even under different battery chemistries. The flat voltage profile and hysteresis are effectively captured by the model due to its ability to learn complex nonlinear and temporal relationships from data. Thus, the proposed method applies not only to typical lithium-ion battery chemistries but also to LFP batteries, provided that the model is trained on representative data covering the full operating range of the target chemistry.

## Conclusion

To address the phenomenon of non-linear battery behaviour and long-term dependency, the proposed hybrid deep learning model with dual optimization is used to estimate the SoC accurately. The proposed method combines the advantages of optimized hyperparameters tuned by optuna and NSGA II with the proposed model KANBiLSTMAtt. KAN model directly learns the nonlinear input, BiLSTM captures the temporal dependencies in both forward and backward directions, and the attention mechanism allows the model to focus on the most informative data. A dual optimization algorithm, optuna and NSGA II used to tune the model hyperparameters and optimize the model size and computational cost. The proposed model was executed with two different datasets. One dataset profiles different driving cycles (DST, FUDS, US06, and BJDST) and another for different temperatures (0 °C, 10 °C, and 25 °C). The SoC estimation is assessed through accuracy measures, including MAE, RMSE, and R2. All the driving cycles in dataset1, RMSE around 0.01, and R2 is 0.99. And for all the temperatures in dataset 2, RMSE is around 0.02, and R2 is 0.98. The proposed method exhibits strong generalization capability and robustness under varying temperatures and dynamic driving conditions. This hybrid model enables fast and reliable real-time SoC estimation using simple forward pass and remains robust under measurement noise without increasing online computational cost. These results show that the suggested method works for estimating the SoC of a battery in real time and suggest that it can be used in electric vehicle (EV) battery management systems (BMS) that work in different environments and usage scenarios. Future work will focus on the deployment of the proposed model for real-time applications, enabling its integration into practical battery management systems for electric vehicles and energy storage solutions.

## Data Availability

Data can be requested from the corresponding author.
